# KIF2C/MCAK a prognostic biomarker and its oncogenic potential in malignant progression, and prognosis of cancer patients: a systematic review and meta-analysis as biomarker

**DOI:** 10.1080/10408363.2024.2309933

**Published:** 2024-02-12

**Authors:** Nina-Naomi Kreis, Ha Hyung Moon, Linda Wordeman, Frank Louwen, Christine Solbach, Juping Yuan, Andreas Ritter

**Affiliations:** a Obstetrics and Prenatal Medicine, Gynaecology and Obstetrics, University Hospital Frankfurt, J. W. Goethe-University, Frankfurt, Germany; b Department of Physiology and Biophysics, University of Washington School of Medicine, Seattle, WA, USA

**Keywords:** KIF2C, MCAK, microtubule dynamics, migration, motility, invasion, metastasis, overall survival, disease free survival

## Abstract

KIF2C/MCAK (KIF2C) is the most well-characterized member of the kinesin-13 family, which is critical in the regulation of microtubule (MT) dynamics during mitosis, as well as interphase. This systematic review briefly describes the important structural elements of KIF2C, its regulation by multiple molecular mechanisms, and its broad cellular functions. Furthermore, it systematically summarizes its oncogenic potential in malignant progression and performs a meta-analysis of its prognostic value in cancer patients. KIF2C was shown to be involved in multiple crucial cellular processes including cell migration and invasion, DNA repair, senescence induction and immune modulation, which are all known to be critical during the development of malignant tumors. Indeed, an increasing number of publications indicate that KIF2C is aberrantly expressed in multiple cancer entities. Consequently, we have highlighted its involvement in at least five hallmarks of cancer, namely: genome instability, resisting cell death, activating invasion and metastasis, avoiding immune destruction and cellular senescence. This was followed by a systematic search of KIF2C/MCAK’s expression in various malignant tumor entities and its correlation with clinicopathologic features. Available data were pooled into multiple weighted meta-analyses for the correlation between KIF2C^high^ protein or gene expression and the overall survival in breast cancer, non-small cell lung cancer and hepatocellular carcinoma patients. Furthermore, high expression of KIF2C was correlated to disease-free survival of hepatocellular carcinoma. All meta-analyses showed poor prognosis for cancer patients with KIF2C^high^ expression, associated with a decreased overall survival and reduced disease-free survival, indicating KIF2C’s oncogenic potential in malignant progression and as a prognostic marker. This work delineated the promising research perspective of KIF2C with modern *in vivo* and *in vitro* technologies to further decipher the function of KIF2C in malignant tumor development and progression. This might help to establish KIF2C as a biomarker for the diagnosis or evaluation of at least three cancer entities.

## 1. Background

Orchestrated regulation of microtubule (MT) dynamics is critical for various cellular functions, including cell division of all eukaryotic cells [1]. During cell division, the MT-based mitotic spindle is required to guide a faithful chromosome separation into two identical daughter cells [2]. The dynamic MT structure consists of α- and β-tubulin polymers and its assembly/disassembly is regulated by a great number of different protein families such as motor proteins, microtubule associated proteins (MAPs) and kinases [1,3]. The microtubule organizing centers (MTOCs) are the main nucleation centers for MTs and are called centrosomes in animal and human cells [4]. These structures typically combine two main mechanisms: the nucleation of novel MT filaments and the anchoring of minus-end MTs, which are essential for the stability of the MT cytoskeleton [5]. The growth and shrinkage phases at the plus MT-ends are driven by two distinct states of MT subunits, the GTP- and GDP-bound tubulin dimers, which have different structural properties, binding kinetics as well as interaction partners [6]. The model of a “GTP cap,” which prevents MT catastrophe, was established by Voter and Erickson in 1984 [7] and was further corroborated by using non-hydrolyzable GTP analogs like GMPCPP [8]. In line with these notions, the degradation of the “GTP cap” leads to the induction of MT catastrophe, which suggests a competition between the growing plus-end, by adding *de novo* GTP-tubulin subunits and the steady hydrolysis of GTP in the MT lattice [8,9]. This steady process of dynamic instability is a key process in spindle assembly, chromosome separation, organization of the cytoskeleton during mitosis and interphase, and the exploration of the extracellular environment (ECV) [6,10]. MT dynamics are vital in these cellular functions, and are orchestrated by various regulators. More than 663 MAPs are identified in eukaryotes, characterized by their ability to bind and interact with the MT lattice, tubulin heterodimers, MT plus-ends and their function to regulate MT dynamics [11,12], including MT stabilizing proteins such as Tau [13] and cytoplasmic linker-associated protein (CLASP) [14], MT modulator RBP-J interacting and tubulin-associated protein (RITA) [15] and depolymerizing proteins including the highly conserved non-motile kinesin-13 family members [16,17].

## 2. Materials and methods

### 2.1. Search strategy

This in-depth systematic review about the prognostic value of KIF2C/MCAK (hereafter revered as KIF2C) follows the “Preferred Reporting Items for Systematic Reviews and Meta-Analyses” (PRISMA) guidelines [18]. Databases used for the article retrieval were PubMed/NCBI, Web of Science as well as the preprint databases bioRxiv and Researchsquare. All databases were scanned with the search strategy shown in Supplemental Table 1 for Pubmed/NCBI or a strategy modified for the individual database. All literature screening was performed by both first authors and the last author of this systematic review.

### 2.2. Eligibility

The inclusion and exclusion criteria were defined prior to data acquisition. This systematic review is focused on studies using KIF2C/MCAK as a prognostic marker in any cancer entity, and includes publications in the English language between 1996 and 2023 (date: 05.06.2023). Abstracts from conferences and reviews were excluded, and pre-prints were intentionally included in the paper acquisition. A detailed description of the screening process can be found in Supplement Figure 1. Specific exclusion criteria concerning cancer subtypes, mutations or any other variations were not considered. The primary readout for all articles was defined as the prognostic value of KIF2C/MCAK gene or protein expression in the overall survival (OS) of cancer patients. The secondary objective was the correlation between KIF2C/MCAK and the disease-free survival (DSF) in all cancer patients, and the tertiary objective was to acquire all other clinicopathological features correlated to the expression of KIF2C/MCAK. Risk and bias calculations were not performed.

### 2.3. Data extraction

The obtained data were extracted into two independent tables. Table 1 includes the correlation between KIF2C/MCAK expression and the clinicopathological features of eleven cancer entities. Table 2 summarizes molecular mechanisms involved in KIF2C oncogenic potential, separated into tumor entities. This systematic review is focused on studies using KIF2C/MCAK as a prognostic marker in any cancer entity. Third the data concerning the OS/DSF and the expression of KIF2C/MCAK was extracted into a meta-analysis visualized in Figure 3 by forest plots portraying the hazard ratio of both clinicopathological features. To prevent a selection bias, all studies were included regardless of their statistical significance. Weighted scores (ws), based on the patient cohort of the studies, were generated and used to calculate the mean OS/DFS value for each meta-analysis.

## 3. Kinesin-13 family

Kinesins are ATP-driven motor proteins with the ability to move across the MT lattice, mostly toward MT plus-ends, gaining the required energy by hydrolysis of ATP subunits [19]. They fulfill multiple functions such as the intracellular transport of macromolecules, vesicles or cell organelles, regulating MT dynamics, MT morphogenesis, chromosome segregation, spindle formation and elongation [20]. The entire kinesin super-family is highly diverse and has been subdivided into 14 distinct families based on phylogeny, structure and sequence homology [21]. While many kinesins are MT polymerizing or stabilizing proteins such as kinesin-5 [22], this superfamily also contains proteins with the ability to induce MT depolymerization, the so-called kinesin-13 family, also known as middle motor domain type (m-type) [19]. Members of the kinesin-13 family depolymerize MTs by using energy from ATP hydrolysis and remove α- and β-tubulin subunits from the MT polymer [23]. Four different members of the kinesin-13 family, namely KIF2A, KIF2B, KIF2C (also known as mitotic centromere-associated kinesin) and KIF24 have been described in the human genome [24]. They have functions in spindle assembly, chromosome segregation, MT-kinetochore attachment, migration and cilia formation [25]. The potent depolymerization activity of the kinesin-13 family depends on the effective direct targeting of MT-ends or by one-dimensional diffusion on the MT lattice [17]. This specific targeting of the MT-end is supported by the positively charged and highly conserved neck domain of the kinesin-13 members [26]. At the MT-ends, these proteins induce MT catastrophe through their enzymatic ability to cause bending of a single tubulin protofilament, leading to the release of α- and β-tubulin dimers [25].

The best described member of the kinesin-13 family is KIF2C [27]. It plays an essential role in the modulation of MT dynamics. KIF2C is found frequently at centromeres, kinetochore MTs and astral MTs [28–30]. The protein has various functions during mitosis, in spindle assembly, chromosome congression, kinetochore-MT attachment and chromosome segregation [31–34]. In addition to its roles during mitosis, KIF2C is important in cytoskeleton remodeling, especially in the regulation of MT dynamics during migration, invasion and focal adhesion (FA) turnover [35–38]. Recently, KIF2C was described to have a negative role in ciliogenesis [39] as well as in DNA double strand break dynamics and repair [40]. KIF2A, is predominantly located at centrosomes where it affects the spindles dynamics and is therefore essential for both bipolar spindle assembly and chromosome movement [24,41,42]. Additionally, KIF2A contributes to pole-directed chromosome movement in anaphase by disassembling MTs at their minus-ends at the spindle poles in association with a poleward MT flux [43].

Interestingly, KIF2A also plays a role in meiotic cells. It has been shown that KIF2A localizes to the meiotic spindle, specifically to the spindle poles and inner centromeres in metaphase, and was translocated to the midbody in telophase. Depletion of KIF2A led to severely defective spindles [44]. Besides its function during cell division, KIF2A is essentially involved in primary cilia disassembly [45] and in cortical neuron migration and differentiation [46]. KIF2B was reported to promote faithful chromosome segregation [47,48]. It is expressed at low levels in almost all human tissue types except testis. [49,50]. KIF24, the fourth member of the family, shares significant homologies with other members of the kinesin-13 subfamily and is involved in MT depolymerization activity in cilium formation [51], clustering of supernumerary centrosomes [52], disassembly and maturation of centrioles [53,54]. Altogether, these data emphasize the crucial roles of the kinesin-13 family in regulating MT dynamics through all phases of the cell cycle. Interestingly, most of these functions are known to be highly deregulated during cancer development and progression.

### 3.1. KIF2C domains, structure, localization and mitotic regulation

**Figure 1.**
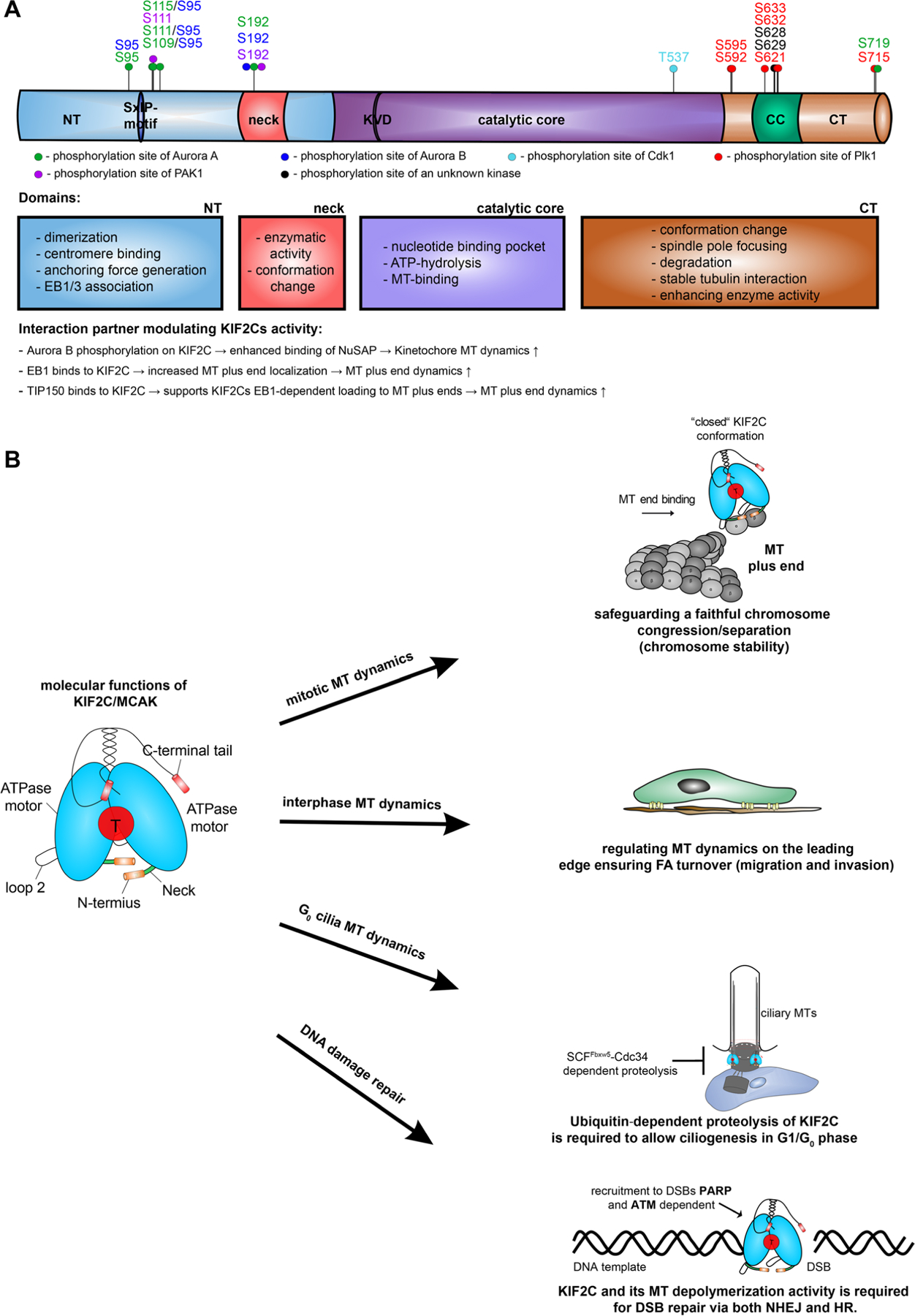
Illustration of KIF2C’s phosphorylation sites, domains, interaction partners and molecular functions. (A): KIF2C’s domains, functions and phosphorylation sites are depicted. The phosphorylation sites are marked as green for Aurora A, blue for Aurora B, light blue for Cdk1, red for Plk1, purple for PAK1 and black for a phosphorylation by an unknown kinase. (B): schematic illustration of KIF2C/MCAK’s molecular involvement in regulating chromosome congression/segregation, FA turnover, ciliogenesis and DSB repair. The figure was modified and updated from [17]. Abbreviations: Cdk1: Cyclin-dependent kinase 1; Plk1: Polo-like kinase 1; PAK1: p21-activated kinase; EB1: end-binding protein 1; FA: focal adhesion; DSB: double-strand break; NT: NH2-terminus; CT: carboxy-terminus; NHEJ: non-homologous end joining; HR: homologous recombination.

The protein structure of KIF2C can be divided into four functional units (Figure 1A). The N-terminal domain contains regions important for dimerization, centromere binding, generation of anchoring forces and the binding to EB1/EB3 with its SxIP motif, while the C-terminal tail regulates KIF2C’s tubulin interaction, stability, conformation and enzymatic activity [17,55]. The stability of KIF2C is mainly regulated by its phosphorylation at S621 on the C-terminus, which facilitates its recognition by the ubiquitin/proteasome dependent APC (anaphase-promoting complex)/C (cyclosome) pathway leading to its D-box dependent degradation in mitosis [29]. Moreover, a study using deuterium exchange mass spectrometry showed that the closed N-terminal form of KIF2C imparts the outward curvature and lateral separation of α/β-MT dimers [56]. KIF2C’s neck region is responsible for its enzymatic activity and conformation changes. Enzymatic regulation is highly dependent on the positively charged neck structure, which can be modulated by several phosphorylation sites including the highly conserved phosphorylation site S192 for multiple kinases, including Aurora A and B [37,57]. The X-ray crystallographic structure of KIF2C with additionally docked tubulin dimers has revealed that the neck and KVD motif of KIF2C are able to directly interact with the α-tubulin distal end [16,58]. The catalytic core domain generates the energy required for MT depolymerization by ATP hydrolysis and is responsible for a robust MT binding [59]. At the C-terminus is a domain that, like the N-terminus, is responsible for the conformational change and stable tubulin interaction [17]. KIF2C functions in a dimeric state, which increases its robust MT depolymerization activity, and the dimeric state also facilitates attachment to MT-ends [26,59–62]. KIF2C interacts directly with MTs on multiple sites, including its helix a4, the loop 2 structure with its KVD finger motif, the loop 8 in the motor domain and the positively charged neck region [58,63,64].

All of these functional domains are regulated by multiple phosphorylation sites, which modulate both the activity and localization of KIF2C, as shown by multiple investigations using phospho-antibodies and site-directed mutagenesis [26,64]. Key players in this phosphorylation events are the mitotic kinases Aurora A/B, Plk1 (Polo-like kinase 1), Cdk1 (Cyclin-dependent kinase 1), and PAK1 (p21-activated kinase 1) [17]. One of the major phosphorylation sites regulating the depolymerization activity of KIF2C is S192, which is phosphorylated by Aurora A/B and PAK1 [37,65,66]. Both phosphorylation and dephosphorylation of this site affect the catalytic activity of human KIF2C in cancer and normal cells [37,57]. Furthermore, interference with this phosphorylation site partly arrests cancer cells in metaphase, and induces congression and segregation defects [37]. In support, Aurora B also phosphorylates the conserved serine S196 in *Xenopus leavis*. This phosphorylation leads to increased localization of KIF2C near the centromere, which downregulates its depolymerization activity, especially in the crucial kinetochore-centromere region [67,68]. Functionally, the Aurora B phosphorylation at S196 in the neck region triggers a change in the conformation of KIF2C and reduces the interaction between the C-terminus and the neck region, which significantly impairs its MT affinity [69]. Additionally, Aurora B regulates the localization of KIF2C’s subcellular localization by phosphorylation of five different sites (S92, S106, S108, S112 and S186) in hamster KIF2C [30]. While the localization of the phospho-mimetic KIF2C 5xE is increased in the centromere region and causes aberrant mitotic spindles, the non-phosphorylatable KIF2C 5xA is localized predominantly in the kinetochore region with reduced binding to TIP150 (tracking protein of 150 KDa) [30]. Like Aurora B, Aurora A targets the same phosphorylation sites and phosphorylates primarily 5 sites (S95, S109, S111, S115, S192) in the N-terminus and neck region of KIF2C [70]. Further, KIF2C is co-localized with nuclear mitotic apparatus (NuMA) protein and *Xenopus* microtubule-associated protein (XMAP215) at the center of Ran-asters, where its activity is regulated by Aurora A-dependent phosphorylation of S196, resulting in proper pole focusing [70]. In addition, the Aurora A phosphorylation of the site S719 increases KIF2C spindle pole focusing ability [70,71]. Another key kinase in the regulation of KIF2C is Plk1, which phosphorylates the site S632/S633 and increases the activity of KIF2C during the early phases of mitosis [72]. Plk1 also phosphorylates S621 of KIF2C without a required priming phosphorylation by another kinase. This phosphorylation facilitates the recognition by the Cdc20 subunit of the APC/C complex, leading to its D-box dependent degradation by the proteasome at the transition from metaphase to anaphase [29]. The fourth important mitotic kinase in regulating KIF2C is Cdk1, which phosphorylates T537 in the core domain of KIF2C and thereby enhances the catalytic activity of KIF2C [73]. Interfering with this phosphorylation generates substantial mitotic defects during chromosome congression and segregation [73]. A T537E mutant disrupts the recognition of the microtubule end, decreasing its capacity to stimulate ADP dissociation at the MT minus-end [74]. Another regulatory mechanism of KIF2C is the binding to MTs, as well as tip tracking proteins such as EB1 and TIP150. Phosphorylation of the neck domain abolishes these interactions, highly reducing KIF2C’s depolymerization activity, especially at the MT plus-ends [34,75]. Additionally, binding to the MAP nucleolar spindle-associated protein (NuSAP) significantly enhances the activity of KIF2C on kinetochore MTs [76]. In summary, these observations highlight the crucial importance of various mitotic proteins in regulating the fine-tuned localization and activity of KIF2C *via* diverse molecular mechanisms during mitosis and beyond.

### 3.2. KIF2C regulation of cell polarization, migration and invasion

The process of cell polarization, migration and invasion is highly dynamic and involves all three filament types of the cytoskeleton: actin microfilaments, intermediate filaments and MTs [77]. Key functions of MTs in these processes are highly complex and include three biological mechanisms. The long-distance intracellular transport of proteins and vesicles, the delivery of new membrane components and signaling molecules essential for the assembly of structures transmitting traction forces and the recycling of adhesion components as well as receptors [77,78]. MTs are physically connected to FA sites, which are specialized structures within the cell, where various integrin receptors interact with the ECM (extracellular matrix) components and the actin cytoskeleton inside the cells [79]. MTs facilitate not only FA assembly by transporting vesicles and structure proteins to the forming FAs, but are also essential for the assembly of FAs by transporting autophagosomes and other components to the retrenching FAs [77,80].

It is therefore hardly surprising that KIF2C has been linked to MT dynamics during interphase, polarization of ECs, assembly and disassembly of FAs and migration/invasion of cancer as well as benign cells [35–37,57]. In detail, KIF2C has been shown to modulate the MT growth speed and branching in compliant 2D and 3D ECM in ECs [81]. Mechanistically, the KIF2C-RAC1 (Rac Family Small GTPase 1) pathway is regulated by Aurora A. The kinase ensures and regulates EC polarization and directional migration by regulating MT dynamics through KIF2C on the leading edge of cells (Figure 1(B)) [35]. In detail, the activity of RAC1 on the trailing edge is able to enhance the kinase activity of Aurora A, which in turn is able to modulate KIF2C’s MT depolymerization activity as shown in mitosis by phosphorylation of S196 [35]. In line with this, the immortalization of human bronchial epithelial cells by expressing K-Ras (Kirsten rat sarcoma virus) and knockdown of p53, resulted in a simultaneous expression of KIF2C and increased the dynamic instability of MTs and enhanced cell migration [82]. Moreover, KIF2C was shown to regulate cell polarity, protrusion formation, centrosome reorientation during migration and the FA turnover by regulating the plus-tip dynamics in human cervical cancer cells and retinal pigment epithelial cells [36,57]. Overall, these reports indicate KIF2C’s involvement in cell migration and invasion of cancer cells [36,37,57,83].

## 4. Altered KIF2C is involved in several hallmarks of cancer

**Figure 2.**
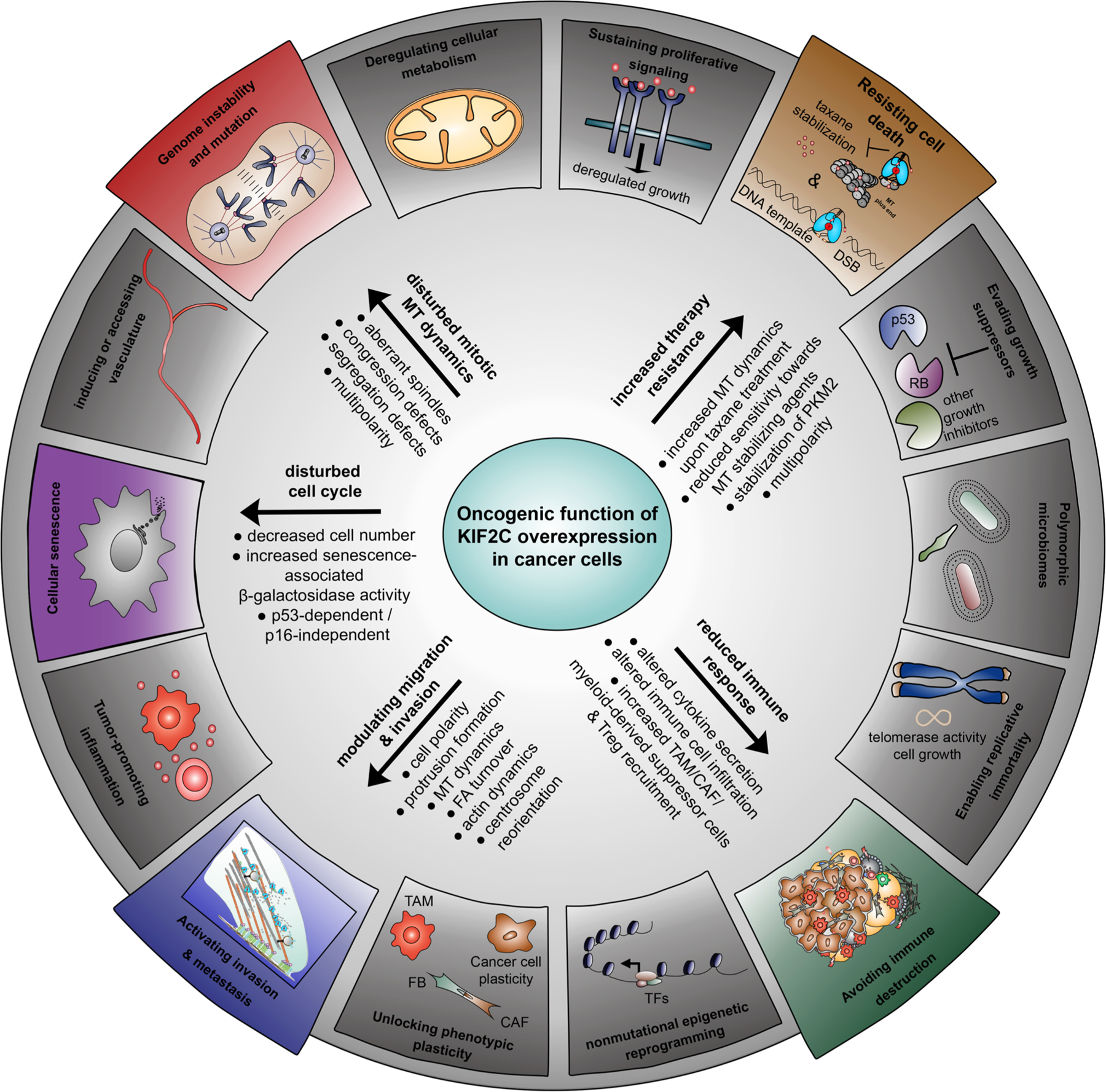
The model depicts how deregulated KIF2C/MCAK is associated with some of the fourteen known and discussed hallmarks of cancer including acquired capabilities and enabling characteristics [84]. These hallmarks encompass the following terms: deregulated cellular metabolism, sustaining proliferative signaling, resisting cell death, evading growth suppressors, polymorphic microbiomes, enabling replicative immortality, avoiding immune destruction, nonmutational epigenetic reprogramming, unlocking phenotypic plasticity, activating invasion and metastasis, tumor-promoting inflammation, cellular senescence, inducing or accessing vasculature and genome instability and mutation. Deregulated KIF2C may be associated with at least five of these hallmarks by promoting migration and invasion, disrupting MT dynamics, impairing the immune response, inducing senescence and increasing therapy resistance to MT interfering agents.

The hallmarks of cancer propose a panel of functional alterations that a cell has to acquire to change its phenotype from a benign cell to a neoplastic growth state and its ability to form malignant tumors [84,85]. These hallmarks include eight characteristics and six capabilities, including (a) genome instability, (b) resistance to cell death, (c) activating invasion/metastasis and (d) avoiding immune destruction, (e) cellular senescence [84,86], all of which can be associated with the deficient function of KIF2C induced by its deregulation (Figure 2) and will be summarized below. This altered expression or activity is either induced directly by an altered gene expression, changed DNA methylation, abnormal mRNA regulation such as long non-coding RNAs (lncRNAs) or micro RNAs (miRNAs), or indirectly by post-translational modifications *via* overexpressed kinases such as Aurora A, Aurora B, Plk1 or Cdk1.

### 4.1. KIF2C, genome instability and mutation

MT dynamics during metaphase and anaphase is the driving force to ensure a flawless chromosome separation, which safeguards genome stability in cells. The kinetochore–MT interface is crucial to prevent congression defects as well as segregation errors [87]. KIF2Cs activity and functions are precisely regulated by various kinases and its malfunction results in aberrant spindles, defective chromosome congression and segregation, leading to an error-prone mitosis, which is associated with chromosomal instability (CIN), a known hallmark of oncogenesis [83]. Indeed, 3T3 fibroblasts expressing a nonfunctional KIF2C demonstrated a high degree of CIN [88]. Interestingly, p53 knockout in these cells reduced proliferation, and mice with KIF2C^deficient^/p53^KO^ xenograft tumors had a highly increased progression-free survival (PFS) compared to KIF2C^WT^/p53^KO^ [88]. Additionally, a recent study showed that inhibitors against KIF2C’s depolymerization activity were sufficient to induce aneuploidy in different cancer cell lines [89], highlighting KIF2Cs, function in safe-guarding chromosome stability during mitosis. In line, Wagenbach et al. reported that slight changes in KIF2C’s expression pattern induces CIN, a weak overexpression as well as a scarce downregulation led in their reporter FKBP-MCAK CRISPR cell model to a significant number of cells with lagging chromosomes in telophase [90]. In summary, these data illustrate how KIF2C’s increased and decreased expression in cancer cells is able to fuel tumorigenesis.

### 4.2. KIF2C and resisting cell death

Interestingly, MTs in recent years have been shown to be directly involved in the DNA damage response in at least three levels: (1) cytoplasmic MTs facilitate the transport of proteins involved in DNA damage repair into the nucleus, (2) MTs are able to modulate the chromatin structure and induce chromatin reorganization, (3) DNA double-strand breaks (DSBs) are mobilized by MTs, which promote the recruitment of DNA repair proteins to these sites [91]. In line with this notation, a recent study indicates that KIF2C is also recruited to DNA damage sites, which was dependent on the activity of poly (ADP-ribose) polymerase 1 (PARP) and Ataxia-telangiectasia mutated (ATM) [40]. Abolishing KIF2C’s activity impaired double strand break repair *via* both non-homologous end joining and homologous recombination [40]. This, in addition to its MT destabilizing function, may explain how KIF2C is involved in resisting cell death induced by known anti-cancer drugs, such as taxanes and doxorubicin, that are known to cause DNA damage [92–94]. This is in line with the high incidence of missense mutations or amplifications in the KIF2C gene in pancreatic adenocarcinoma [95] or the mutation of E403K in KIF2C found in colorectal cancer patients [96].

### 4.3. Activating invasion and metastasis

Since the process of cell migration is highly dependent on both actin filaments and MTs [97], it is not surprising that multiple studies highlight the importance of KIF2C in migration, invasion and metastasis [36,37,57,98,99]. Mechanistically, the first function of KIF2C during these processes is to facilitate the modulation of the cell polarization by fostering dissimilarities in MT dynamics between the leading and trailing edges of cells [35]. This process is highly dependent on an Aurora A-KIF2C-RAC1 signaling pathway, in which Aurora A finely tunes KIF2C’s depolymerization activity in the growing MTs in the interphase [35,57]. Secondly, the centrosome repositioning is highly dependent on active MT dynamics in migrating cells. Knockdown of KIF2C was associated with defects in centrosome repositioning in retinal pigment epithelial cells (RPE-1) [57], which disturbs the MT dynamics required to form a leading and trailing edge in migrating cells. Third, the orchestrated assembly and disassembly of focal adhesions requires MTs for endo- and exocytotic processes [100]. Interfering with the expression of KIF2C leads to severe defects during FA turnover [57] and deregulated phosphorylation events of key FA proteins including paxillin and focal adhesion kinase (FAK) [36]. Fourth, the polymerization of the actin filaments and MTs is interdependent. Interfering with the plus-tip dynamics in the interphase by knockdown or overexpression of KIF2C highly reduced the polymerization of actin filaments [36]. These data underscore the key roles of MAPs during cell migration, especially of KIF2C in cell motility of cancer cells.

### 4.4. Avoiding immune destruction

The recognition of malignant cells and the subsequent destruction of malignant cells is a key barrier to tumor formation and progression [86]. As a consequence, malignant tumors form an immunosuppressive tumor microenvironment (TME) [101]. In this complex process, there is evidence indicating that KIF2C could be associated with the immune response. Studies in hepatocellular carcinoma (HCC) [102], glioma [103,104], kidney renal clear cell carcinoma (KIRC) [105] and pancreatic ductal adenocarcinoma (PDAC) [99] suggest that a high expression of KIF2C interferes with proper immune cell infiltration by recruiting increased numbers of tumor-associated macrophages, cancer-associated fibroblasts, myeloid-derived suppressor cells and Treg into the TME [95]. The disturbed immune response was connected with decreased levels of immunomodulatory IL18 and IL1β based on RNA sequencing and protein analyses of PDAC samples [99]. Further, KIF2C was shown to be involved in the complement and coagulation cascade, cytokine-cytokine receptor interaction, and IL17 signaling pathway by GO and KEGG pathway analyses of kidney renal clear cell carcinoma (KIRC) and liver hepatocellular carcinoma [105], suggesting an interesting new function for KIF2C. This could be related to its role in intracellular trafficking and involvement in endocytosis, though further investigation is required [36]. Additionally, KIF2C is able to negatively regulate ciliogenesis, which is a cell organelle shown to be involved in cell-cell signaling, immune cell function and immune cell metabolism [106]. Deregulated degradation or overexpressed KIF2C causes an accumulation on the basal bodies in the following G_1_/G_0_ phase and reduced ciliogenesis [107]. This results in broad cellular consequences such as decreased signaling of the canonical hedgehog pathway, a pathway exclusively mediated by the primary cilium, which has implications for cell fate and self-renewal [107,108].

### 4.5. Cellular senescence

Cellular senescence is known as a protective mechanism for maintaining a controlled tissue homeostasis and serves as a complementary mechanism to cellular apoptosis [84]. Both senescence and apoptosis should help organisms to remove damaged, dysfunctional or unnecessary cells [109]. However, several decades after the discovery of senescent cells, a multitude of studies showed that the malignancy of cancer cells is fueled by senescent cells [110]. Intriguingly, KIF2C was shown to be involved in the induction of cellular senescence in human primary cells (dermal fibroblasts and umbilical vein endothelial cells) [111]. Knockdown of KIF2C induced cellular senescence that was highly dependent on p53 but was not associated with the known senescence regulator p16 [111]. In line with this observation, a recent study could show that *KIF2C* together with four other cellular senescence-related genes, is associated with poor prognosis of patients with idiopathic pulmonary fibrosis [112].

In summary, these observations clearly support the concept that KIF2C is involved in at least five different hallmarks of cancer, suggesting a possible role in tumor development, progression and malignancy.

## 5. Molecular mechanisms involved in KIF2C’s oncogenic potential in multiple cancer entities

Numerous studies support KIF2C’s oncogenic potential showing that it is highly expressed in tumor cells from different origins and that the degree of de-differentiation of tumor cells depends on the expression level of KIF2C [95,113–117]. This increased expression was also found in two independent studies at the mRNA level in the entire patient sample cohort of the TCGA (The Cancer Genome Atlas) database including 33 different cancer entities [95,118]. KIF2C’s role in multiple vital functions and its high protein/gene expression will be discussed in various cancer entities including breast cancer, non-small lung cancer, hepatocellular carcinoma and gastric cancer, highlighting its crucial role in carcinogenesis.

### 5.1. Breast cancer

**Figure 3.**
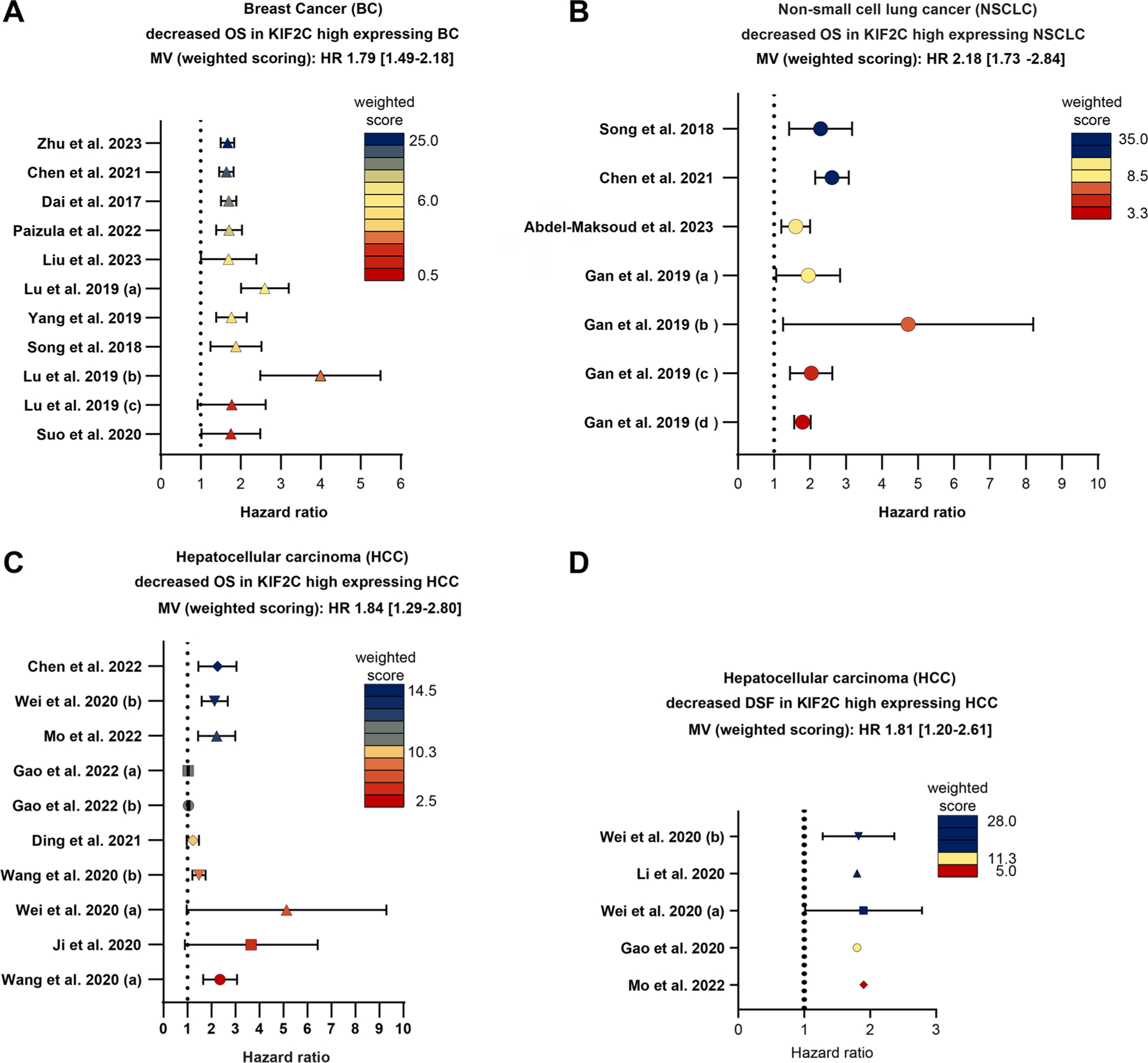
High expression of KIF2C is associated with reduced OS and DSF in BC, HCC and NSCLC. (A–C) The meta-analyses concerning KIF2C/MCAK’s importance as a prognostic marker is depicted as forest plots for OS of BC, NSCLC and HCC patients (A–C). (D) Meta-quantification of KIF2C/MCAK involvement in DSF in HCC patients. All individual studies were scored based on their patient cohort. The weighted score (ws) is visualized in a heatmap, dark blue (high ws), yellow (intermediate ws) and red (low ws). The overall mean value (MV) extracted from all studies was calculated by integrating the weighted score. Abbreviations: OS: overall survival; DFS: disease-free survival.

Breast cancer represents the best studied cancer entity correlated with KIF2C’s expression, prognostic value and involvement in therapy resistance (Figure 3(A) and Table 1). The first studies were conducted almost two decades ago, where Nishidate and colleagues identified KIF2C as one of 34 genes that were expressed differentially in breast tumors with lymph node metastasis compared to tumors without metastasis [119]. Moreover, two *in vitro* studies showed that protein regulator of cytokinesis 1 (PRC1) and KIF2C formed a functional complex and that both were upregulated in several breast cancer cell lines [120,121]. Knockdown of either PRC1 or KIF2C inhibited proliferation of multiple breast cancer cell lines, which was associated with morphological changes especially during mitosis, in these cancer cells [121]. The gene level of *KIF2C* was found to be correlated with poor outcomes in breast cancer patients [122]. Moreover, the gene methylation of *KIF2C* was significantly elevated in luminal A breast cancer samples with prognostic value, together with three other cell cycle and proliferation regulators Ki-67, UBE2C (ubiquitin conjugating enzyme E2C) and HDAC4 (histone deacetylase 4) [123]. A bioinformatic multi-layer interference approach of context-dependent gene networks revealed that KIF2C was a target gene for ER^−^/HER2^−^ (estrogen/human epidermal growth factor receptor 2) breast cancers and its gene expression was positively regulated by E2F1 (E2F transcription factor 1), which has crucial roles in cell cycle control [124,125]. This was continued by three other bioinformatic approach studies [126–128], which displayed that a four gene panel consisting of *KIF2C*, *KIF1A*, *FAM134B* (Reticulophagy Regulator 1) and *ALCAM* (Activated Leukocyte Cell Adhesion Molecule) had diagnostic translational potential [126]. This four gene panel was further shown to have implications in several hallmarks of cancer, including enabling replicative immortality, sustaining proliferative signaling, activating invasion and metastasis, resisting cell death and deregulating cell metabolism that are connected to breast cancer heterogeneity [126]. Moreover, the expression of *KIF2C*, *PLK1* and *MAD2L1* (mitotic arrest deficient 2 like 1) was correlated to reduced overall survival (OS) in invasive ductal breast carcinoma [127]. In basal-like breast cancer, KIF2C is a crucial hub gene, together with nine other genes [128]. These data were corroborated by a study analyzing the cohort of the cancer genome atlas [129]. It was found that a high expression of KIF2C correlated indeed with poor OS and, vice versa, patients with a low expression of KIF2C had significantly more favorable OS [129,130]. To further support these findings, the expression of KIF2C was found to be up-regulated in all breast cancer subtypes compared to normal tissue, with the triple negative breast cancer (TNBC) subtype expressing the highest level of KIF2C. To further support these findings, the expression of KIF2C was found to be up-regulated in all breast cancer subtypes compared to normal tissue, with the triple negative breast cancer (TNBC) subtype expressing the highest level of KIF2C. Corroborating these data, five genome sequencing sets consisting of 63 TNBC samples and 169 non-TNBC samples were analyzed and five hub genes *TPX2* (Targeting protein for Xklp2), *CTPS1* (CTP Synthase 1), *KIF2C*, *MELK* (maternal embryonic leucine zipper kinase) and *CDCA8* (cell division cycle associated 8) were again identified [131]. In agreement, it was found that KIF2C expression together with three other genes was correlated with a reduced regression free survival and highly expressed KIF2C was connected to earlier relapse in TNBC patients [132]. Interestingly, a screen of exclusively metastatic breast cancers revealed similar data [133]. Again, KIF2C was identified among five important hub genes *TPX2*, *CDCA8*, *BUB1B* (Budding Uninhibited by Benzimidazoles 1 Homolog Beta), *CCNA2* (cyclin A2) and *KIF2C* associated with a significant enhanced risk for distant metastasis [133]. These data were further consolidated by seven recently published studies, which corroborated the valuable role of KIF2C as a biomarker correlated with poor prognosis for all breast cancer subtypes [130,131,134–138] (Figure 3(A), OS; mean HR 1.79 [1.49–2.20]). Interestingly, KIF2C^high^ breast cancer tissue was significantly correlated with increased stroma cells [130]. This could be associated with an increased population of cancer-associated fibroblasts, which are also related to poor prognosis of patients with luminal breast cancer subtypes [139].

In addition to its role as a prognostic marker, KIF2C has been reported to be linked to therapy resistance to taxanes [89,93,94] and doxorubicin (DOX) [92]. It was shown in malignant (MDA-MB-231, HeLa and HuH7) and nonmalignant (RPE-1 and CHO) cell lines that taxane resistant cells changed their MT environment and recruited more septin9_i1 and septin9_i3 from actin fibers to MTs, which led to tubulin tyrosination and long polyglutamylated chains. This increased the recruitment of MT destabilizing proteins such as KIF2C and CLIP170, resulting in increased MT dynamics and a reduced sensitivity toward MT stabilizing agents [93,94]. Furthermore, KIF2C was able to directly bind pyruvate kinase M2 (PKM2). This prevented the ubiquitination of PKM2 and its proteasomal degradation. The increased levels of PKM2 promoted autophagy and glycolysis, both mechanisms known to enhance DOX resistance [92]. In conclusion, KIF2C increases breast cancer tumorgenicity, malignancy and is sufficient to induce chemoresistance by multiple mechanisms. As a consequence, KIF2C is regarded as a potential cancer therapy target. In fact, several studies showed that depletion or knockdown of KIF2C interferes with proliferation, migration and invasion capacity of cancer cells [36,37,116,121]. A recent analysis of breast cancer samples integrated in The Cancer Genome Atlas (TCGA) revealed that KIF2C, along with two cell cycle master regulators CCNB1 (cyclin B1) and Plk1, is a promising target for breast cancer therapy [140]. In line with this notion, three microRNAs (miR-10b-5p, miR-485-5p and miR-181c) targeting KIF2C were described [116,117], leading to a reduced gene expression. A high expression of these three microRNAs was positively correlated with patient survival [117]. Interestingly, a recent study found three potent inhibitors against the depolymerization activity of KIF2C [89]. These compounds were able to reduce the clonogenic survival rate of TNBC cells and one compound was able to sensitize TNBC cells to paclitaxel [89]. In summary, these reports corroborate KIF2C as a prognostic biomarker and novel compounds enable KIF2C to be a potential target for breast cancer therapy.

### 5.2. Hepatocellular carcinoma (HCC)

The involvement of KIF2C in HCC was extensively studied *in vitro* and *in vivo* during the last decade [141–143]. These investigations could show that KIF2C modulates the proliferation, migration, metastasis and tumor growth *in vitro* and in a mouse model [141–143]. Additionally, KIF2C affected the stemness of HCC cells by interfering with several key pathways including mTORC1 (mammalian target of rapamycin complex 1) [144], Ras/MAPK (rat sarcoma/mitogen-activated protein kinase) [98], Wnt/β-catenin (wingless/beta-catenin) [144] and MEK/ERK (mitogen-activated protein kinase kinase/extracellular signal-regulated kinases) [145]. Specifically, it was shown that KIF2C is a downstream target of Wnt/β-catenin signaling, leading to its transcriptional activation [144]. In turn, KIF2C enhanced the mTORC1 activity by directly binding to TBC1D7 (Tre2-Bub2-Cdc16 domain family member 7), which interfered with the stability of the mTORC1 inhibitory TSC1-TSC2 complex [144]. This is an essential step in the progression of HCC, since the mTORC1 activity was shown to stimulate cell growth, metabolic re-programming, proliferation and inhibition of apoptosis in HCC patients [146]. In accordance with this, another study could show that the ANLN (anillin actin-binding protein)-KIF2C signaling axis promoted bone metastasis of HCC by activating mTORC1 [147]. Moreover, knockdown of KIF2C in HCC cells gave rise to the transcriptional upregulation of the PI3K/Akt (phosphatidylinositol 3′-kinase/protein kinase B) and MAPK signaling pathways [98], two key pathways in the progression of HCC [148]. At a functional level, KIF2C was shown to promote cell proliferation, migration, invasion, cell cycle progression and inhibited apoptosis as well as induced the epithelial-to-mesenchymal transition (EMT), possibly by the activation of the Ras/MAPK and PI3K/Akt pathways [98]. In support of these observations, knockdown of KIF2C decreased the expression of important EMT transcription factors and downstream targets, including Snail and vimentin [145]. Additionally, the silencing of KIF2C was also associated with decreased p-MEK and p-ERK signals, reducing proliferation, migration and invasion capacity of the epithelial breast cancer cell line HCC1954 [145]. These findings indicate KIF2C’s multifaceted roles in HCC progression, in addition to its known function as regulator for MT stability as well as dynamics to safe-guard chromosome separation during mitosis [17,83].

Consistent with the outlined molecular mechanisms of how KIF2C fuels the malignancy of HCC cells, a large number of investigations have been able to correlate highly expressed KIF2C in HCC patients with several clinicopathological characteristics (Figure 3(C) and Table 1). Twelve different studies indicate that the high expression of KIF2C is associated with a significantly reduced overall survival [98,102,141,142,144,145,149–154] (Figure 3(C), OS; mean HR 1.84 [1.29–2.80]). Furthermore, KIF2C expression was correlated to increased tumor grading [142,153], staging [98], RFS [98,150], DFS [98,141,144,151,153] (Figure 3(D), DSF; mean HR 1.81 [1.20–2.61]), differentiation of the tumor [144], relapse [144], and immune cell infiltration [102]. Consequently, based on diverse bioinformatical approaches, KIF2C was found to be a key hub gene for progression of HCC with its broad involvement in various “Gene Ontology” (GO) and “Kyoto Encyclopedia of Genes and Genomes” (KEGG) pathways containing mitotic nuclear division, nuclear division, chromosome segregation, mitotic sister chromatid segregation, mitotic spindle, spindle pole and kinetochore [152]. Additionally, KIF2C forms interaction networks with other cell cycle master regulators such as *CCNB1*, *CDC20*, *CENPM* (centromere protein M), *TOP2A* (DNA topoisomerase II α), *MYBL2* (Myb-related protein B), *PLK1*, *UBE2C* and *BIRC5* (survivin) [155]. A recent study utilized the protein and gene expression of KIF2C and RAC1 in HCC tissue to characterize a specific HCC patient subtype, which significantly correlated with worse prognosis, clinicopathological grade, increased tumor mutation burden, higher CD8+ T cell infiltration and an altered chemotherapy drug sensitivity [156]. Therefore, KIF2C has been suggested as a prognostic biomarker [149,152–154,156–160] and as a treatment target for HCC [141,144,155].

### 5.3. Non-small cell lung cancer (NSCLC)

In non-small cell lung cancer (NSCLC) tissues, the gene expression of KIF2C was found to be significantly upregulated compared to paired non-tumor samples in three independent RNA sequencing datasets [161]. Moreover, a high expression of KIF2C has been associated with advanced TNM stage in NSCLC patients, especially in the most important and common type of lung adenocarcinoma, and consequently with a dramatically decreased OS (Figure 3(B) and Table 1) [161,162]. These results could be confirmed in several NSCLC cell lines and IHC samples at the protein level, which was again correlated with reduced OS, higher primary tumor staging (T stage), worsened differentiation status and lymph node metastasis [163]. KIF2C knockdown inhibited proliferation, colony formation, migration and invasion of NSCLC cell lines [163]. Interestingly, KIF2C may be targeted by microRNA-325-3p, microRNA-34a-5p and microRNA-186-3p observed *via* a luciferase reporter assay or transcription factor-miRNA-mRNA network computing [163–165]. In accordance with this finding, microRNA-325-3p is considered as a tumor suppressor and its expression was decreased in NSCLC samples [166]. It could be shown that the expression of KIF2C and miR-325-3p were inversely correlated in the 30 NSCLC samples [163]. In line, five recently published bioinformatic studies could identify KIF2C as key signaling hub gene in the progression of lung cancer [164,167–170]. Three of them verified the significantly reduced OS associated with a high expression of *KIF2C* [164,168,169] and all five studies suggested various hub genes, including *KIF2C* as prognostic biomarker and therapeutic target [167–170] (Figure 3B, OS; mean HR 2.18 [1.73–2.84]). Interestingly, this study found multiple drugs, which either interacted with KIF2C or had the potential to regulate KIF2C [164]. Additionally, KIF2C^high^ tissue expression was negatively correlated with the infiltration of CD4^+^ and CD8^+^ T cells [164,169]. Functionally, KIF2C could be shown to promote proliferation, migration and invasion of different NSCLC cell lines and to reduce apoptosis [165]. These effects were likely mediated by the activation of the Akt pathway, as overexpression of KIF2C increased the phosphorylation level of p-Akt, its downstream targets p-GSK-3β and β-catenin [165], which are known for their roles in cancer migration and invasion [171]. The upregulation of KIF2C is likely associated with the hypomethylation of its promotor region [164]. In conclusion, these data highlight that KIF2C is an important protein with oncogenic potential for NSCLC progression involved in metastasis, differentiation, staging and OS.

### 5.4. Glioma

The first report of KIF2C’s association with the malignancy of gliomas was published by Bie and colleagues in 2012 [172]. Their work indicated that *KIF2C* gene and protein expression was related to glioma grading and patient OS. Mechanistically, the expression level of KIF2C was strongly correlated with the proliferation marker Ki-67 [172]. This unfavorable relationship between the expression of KIF2C and the OS of glioma patients was further reinforced by three reports showing that KIF2C was an important hub gene in the overall TCGA glioma dataset [173], low-grade gliomas [174] and secondary glioblastomas [175]. Moreover, two recent *in silico* investigations strengthened the role of KIF2C as key hub gene [103,104], verified its association with poor survival rates in multiple glioma grades (2–4) and a disturbed immune infiltration [103], indicating its diagnostic and prognostic value.

### 5.5. Esophageal squamous cell carcinoma (ESCC)

Based on immunohistochemical staining of malignant tissue sections, a high expression of KIF2C increased the incidence of a high pathologic tumor and poor tumor differentiation status in male patients, though not in female patients [176]. Among patients with similar pathological tumor node metastasis stages, the prognosis was worse in male patients with KIF2C^high^ expression. The OS and DFS were significantly shorter in male patients with high KIF2C expression compared to female patients with high or low KIF2C expression. Its high expression was linked to a significantly increased risk of a higher pathological tumor status and poorer tumor differentiation. The KIF2C expression in ESCC seemed to serve as an independent prognostic marker for male, but not for female patients. Since KIF2C is highly expressed in testis, Duan and colleagues speculate that KIF2C might require an androgen rich environment to carry out its full function, explaining the sex-biased results [176].

### 5.6. Gastric cancer

The gene expression of KIF2C was reported to be significantly higher in gastric cancer tissues compared to its expression in nonmalignant tissues [95,177]. Furthermore, overexpression of KIF2C resulted in significantly higher lymphatic invasion, lymph node metastasis, serosal invasion and a reduced patient survival [113]. As a result, KIF2C^high^ gastric cancers correlated with a poor patient diagnosis with reduced OS, DSS and PFI [177] (Table 1). *In vitro* studies showed that overexpression of KIF2C enhanced cell migration and proliferation with a correlation between the high levels of KIF2C and multiple proliferation genes (*CCN2A*, *CCNB1*, *CCNB2*, *CCNE1*, *CDK1* and *CDK2*) in gastric cancer tissues [113,177]. Another study showed that KIF2C antigen peptides could be used to lyse gastric and colon cancer cells in an HLA class I, and CD8-restricted manner [178]. Moreover, knockdown of KIF2C reduced cell proliferation of gastric cancer cells (GES-1, AGS, MKN-45, NCI-N87, and SNU-1) [177]. These reports suggest that KIF2C may be a clinical marker that correlates with immune cell infiltration and may prove to be an effective therapeutic target in the treatment of patients with KIF2C overexpressing gastric cancer.

### 5.7. Pancreatic cancer (PCa)

The first evidence that KIF2C may be involved in the progression and poor prognosis of PDAC patients was obtained using single-cell RNA-sequencing (scRNA-seq.) [95]. The pan-cancer scRNA-seq. data displayed that KIF2C expression is highly enriched in CD4+ T cells, fibroblasts, NK cells, and most significantly in malignant cells in a comprehensive PDAC cohort (24 primary PDAC tumors and 11 control pancreases) [95]. Interestingly, 1.8% of all patients in the TCGA cohort including PAAD (Pancreatic Adenocarcinoma) had genetic alterations in the KIF2C gene, which included predominantly missense mutations or amplifications [95]. The KIF2C gene and protein expression were enhanced in PAAD. Increased levels of KIF2C expression were significantly associated with poor OS and DSF. High KIF2C was significantly associated with E2F, EGFR, MYC, TP53 and KRAS [95,179]. In support of this, two recent studies with pancreatic cancer (PCa) and two with PAAD found that KIF2C was highly upregulated in pancreatic cancer tissues and correlated with poor patient prognosis and survival [99,179–181]. Specifically, KIF2C^high^ PDACs were significantly correlated with reduced OS, DFS, PFS and postoperative survival time (PST) [99]. Moreover, PCa samples with a high expression of KIF2C reduced patient OS, DFI, DSS and PFI [181]. These clinicopathological features in KIF2C^high^ PCas is likely associated with an altered immune cell infiltration, TME, immune checkpoint activation and a MAPK signaling pathway inhibitor resistance [181]. The expression levels of KIF2C were inversely linked with a reduced methylation level of its DNA [181]. Additionally, upregulated KIF2C was associated with high tumor staging (IV), shorter OS and poorer differentiation [99]. Functionally, the overexpression of KIF2C was shown to be associated with enhanced migration, invasion and colony formation capacity in PCa cells lines ASPC-1 and MIA-PaCa2 [99]. Moreover, these cells had decreased interleukin-1β (IL1β) and IL18 levels, and upregulated CDC20. Finally, a study with a pancreatic xenograft mouse model with MIA-PaCa2 cells reported that a downregulation of KIF2C inhibited the formation of subcutaneous tumors and lung metastasis [99]. In line with a deregulated cytokine secretion, KIF2C expression was also associated with a relative abundance of tumor-infiltrating lymphocytes (TILs) and CD4^+^ T cells in PAAD patients [180]. In further support, several KIF genes including KIF2C, KIF4A, KIF11, KIF14, KIF15, KIF16B, KIF20A, KIF22 and KIF25 were core enriched in the immunologic signature of PAAD samples [179]. Overall, these data strengthen the crucial role of KIF2C in progression of PCa and suggest an involvement in regulating the expression of important immunomodulatory cytokines as well as fueling the malignancy of the cancer cells.

### 5.8. Renal cell cancer (RCC)

In order to find out prognostic genes, a gene analysis was performed using 533 RNA samples of KIRC from the TCGA. KIF2C and two other members of the KIF-family (KIF23 and KIF4A) were found as prognostic genes in KIRC [182]. The study also examined KEGG pathways that have impacted OS of patients with KIRC. These included the cell cycle, homologous recombination and the p53 signaling pathway [182]. KIF2C was implicated in the outcomes of KIRC patients, although no correlation of KIF2C with any clinicopathologic features of KIRC was shown. A further study suggested that long non-coding RNAs (lncRNAs) of *TTK*, *CENPE*, *KIF2C*, *BUB1*, and *RAD51AP1* (RAD51 Associated Protein 1) could act as potential biomarkers for chromophobe renal cell carcinoma progression and prognosis [183]. Indeed, by analyzing the enormous TCGA and gene expression omnibus database, four other studies found that high expression of KIF2C was significantly correlated with reduced OS and DSF in patients with KIRC [95,105,118,184]. Moreover, the expression of KIF2C was significantly associated with infiltrated immune cells such as B cell, CD8^+^ T cell, CD4^+^ T cell, myeloid-derived suppressor cells, Tregs cells and non-immune cells including cancer-associated fibroblasts [105]. The authors suggest that KIF2C is a prognostic biomarker linked to immunosuppression and an interesting target for immunotherapy [105]. Interestingly, a recent report showed that the DNA of KIF2C is significantly hypomethylated in RCC [185]. This is in line with an interesting hypothesis that altered DNA methylation preferably occurs during early carcinogenesis, which is a hallmark of ccRCC [186]. These findings reveal that KIF2C might be a promising biomarker in patients with RCC, as it is frequently deregulated in this cancer entity and correlates with poor prognosis and an immunosuppressive TME.

### 5.9. Colorectal cancer

Similar to gastric cancer, KIF2C was found to be overexpressed in colorectal cancer tissues from 195 patients when compared to the corresponding normal tissue [114,115]. The overexpression was associated with increased lymph node metastasis, venous invasion, lymphatic invasion, peritoneal dissemination, and Dukes’ staging classification, leading to a poor survival of colorectal cancer patients with KIF2C^high^ expression [114]. This was recently corroborated by two independent studies [187,188]. While the first identified upregulated KIF2C as one of four key proto-oncogenes in a comprehensive transcriptomic study of colorectal cancer tissue stages I-IV, adenocarcinoma and mucinous adenocarcinoma [188]. The second included KIF2C into a three gene prognostic signature together with poly (ADP-ribose) polymerase 1 binding protein (PARPBP) and kinetochore-localized astrin/SPAG5 binding protein (KNSTRN) based on a machine learning based approach [187]. These signatures were able to accurately predict the clinical outcomes of stage IV CRC patients, correlated with an increased cancer stem cell phenotype in these patients [187]. Another study used a computational prediction of disease-associated non-synonymous polymorphism analysis to filter out pathological mutations in a large pool of datasets [96]. Their analysis found that the mutation of E403K in KIF2C, which interferes with protein conformation and stability, was associated with the development of colorectal cancer [96]. These observations underline the crucial importance of KIF2C in the progression of colorectal cancer, which might be a possible treatment target by using an antigen specific immunotherapy to stimulate the immunosuppressive environment by triggering a spontaneous CD4^+^ T cell response of the Th1-type in colorectal cancer [115].

### 5.10. Endometrial cancer (EC)

KIF2C was found to be highly upregulated among 344 other genes differentially expressed in endometrial cancer [189]. By generating a protein-protein interaction network, 15 genes were identified with a highly prognostic potential including KIF2C [189]. This is in line with two other studies, which also generated hub gene networks for EC with similar genes containing KIF2C [190,191]. These analyses underscore the notion that the expression of KIF2C is negatively associated with the OS of EC patients [189–191] and is correlated with a poor differentiation status in uterine corpus endometrial carcinoma [191]. Interestingly, on a functional level, KIF2C was shown to stimulate the proliferation, migration and invasion of two endometrial cancer cell lines (Ishikawa and RL95–2) [192]. Additionally, knockdown of KIF2C in a xenograft mouse model significantly reduced the tumor growth and the expression level of KIF2C was negatively correlated to CD8^+^ T cell invasion [192], implicating KIF2C’s role in recruiting immune cells in the TME, important in prognosis and in potential EC intervention.

### 5.11. Ovarian cancer (OC)

The data of KIF2C in OC are inconclusive and illustrate that a fine-tuned expression of KIF2C is highly associated with the patient outcome. In an analysis of a dataset of 396 OC samples and 54 controls, upregulated KIF2C was detected as one of 12 hub genes for OC progression in a protein interaction network, and its expression was negatively associated with poor OS of patients with epithelial ovarian cancer [193]. Another bioinformatic approach found a regulatory network between the upstream transcription factors *SMAD4* (SMAD Family Member 4), *NFKB1* (Nuclear Factor Kappa B Subunit 1), *SMAD3*, *TP53* (Tumor Protein P53), *HNF4A* (Hepatocyte Nuclear Factor 4 Alpha) and their potential downstream targets *KIF2C*, *STAT3* (signal transducer and activator of transcription 3) and *BUB1* [194]. Intriguingly, the authors showed that KIF2C was downregulated in the platinum resistant ovarian cancer cell line A2780 [194]. *FOXM1* was proposed to be an alternative upstream regulator and a transcriptional activator of KIF2C, a transcriptional activator that binds to the KIF2Cs promotor and is associated with paclitaxel resistance, consequently, silencing FOXM1 resulted in downregulation of KIF2C [195]. Ni and colleagues reported in their discussion that low expression of KIF2C was correlated with decreased survival and DSF of OC patients [196]. By contrast, another study showed that KIF2C is upregulated in OC samples, together with nine other hub genes, but could not confirm a significant impact on OS [197]. These studies show that the regulation of KIF2C must be fine-tuned and that its overexpression as well as downregulation are associated with the pathogenesis of OC.

### 5.12. Acute lymphoblastic leukemia (ALL)

While KIF2C has been considered a promising novel biomarker and prognostic marker for various solid tumors, its role in leukemia is hardly defined. One study showed that KIF2C was overexpressed in patients with relapsed acute lymphoblastic leukemia (ALL) [198]. Moreover, this study displayed a correlation between the expression of KIF2C [198] and its binding partner KIF18B [199], together both proteins are associated with a risk of relapse in ALL [198]. Mechanistically, they found that a functional KIF2C was necessary for the accurate lymphopoiesis in zebrafish embryos, which share 50% structural homology to KIF2C [198]. The dependence of hematopoietic stem cells survival on high expression of KIF2C/KIF18B may be a possible pathomechanism of the occurrence of ALL relapse [170].

### 5.13. Other cancer entities

Several single reports or dataset analyses deal with the expression of KIF2C in cervical cancer, nasopharyngeal carcinoma (NPC), laryngeal squamous cell carcinoma (LSCC), laryngeal squamous-cell cancer and bladder cancer [184,200–204]. In cervical cancer, the authors showed that KIF2C mutation is strongly associated with the survival rate, and that KIF2C expression was significantly upregulated in cervical cancer tissues and cervical cancer cells [184]. KIF2C enhanced cell proliferation, invasion, and migration *in vitro* and increased tumor growth *in vivo*. KIF2C knockdown promotes the activation of the p53 signaling pathway. A rescue assay with KIF2C and p53 double knockdown partially reversed the inhibitory influence of KIF2C silencing on cervical cancer processes [184]. The study suggested that KIF2C might be a novel therapeutic target for cervical cancer. Zuo et al. highlighted the role of KIF2C in NPC, with their data indicating that KIF2C is aberrantly overexpressed in multiple mRNA datasets of NPC [200]. Additionally, the knockdown of KIF2C in HNE-1 and CNE-1 NPC cell lines led to reduced motility and migration, increased mitotic defects and an enhanced paclitaxel sensitivity, indicating the broad role of KIF2C in NPC [200]. In LSCC, KIF2C was found to be a hub gene in two independent bioinformatic approaches using multiple comprehensive publicly available RNA expression datasets [201,202], but further investigations are required to decipher the clinicopathological impact of KIF2C’s expression in this tumor entity. Finally, a study indicated KIF2C as a key hub gene in bladder cancer. In their relatively small patient cohort, they could not correlate a high expression of KIF2C with the OS [203]. In support, Yang et al. found that circular RNA circRGNEF promoted bladder cancer progression *via* the miR-548/KIF2C signaling axis [204]. They found that the overexpression of KIF2C was associated with increased invasion, migration and proliferation in the bladder cancer cell lines T24 and UM-UC-3 [204]. In sum, all of these studies point to the notion that KIF2C is associated with increased cancer malignancy in diverse cancer entities.

## 6. Conclusion

In this systematic review, we have highlighted the current data concerning KIF2C’s molecular working mechanisms and its potential involvement in diverse signaling pathways. Further, its association with at least five hallmarks of cancer was emphasized, explaining its oncogenic potential. In line, we have reviewed its expression in various cancer entities and discussed its potential clinical significance, including a meta-analysis correlating KIF2C’s expression with the OS of BC, NSCLC and HCC patients. The data show that the gene expression of KIF2C is significantly increased in at least 33 different cancer types compared to benign tissue [95,118]. Upregulated KIF2C is involved in multiple cellular activities, including proliferation [17], migration and invasion [36], DNA repair and therapy resistance [92] as well as cell-cell and cell-ECM signaling by modulating the primary cilium [39]. The data also suggest that KIF2C^high^ gene or protein expression in cancer tissues could be a diagnostic marker for a number of cancer entities and a prognostic marker for patient OS and PSF. This highlights that a precisely regulated expression and activity of KIF2C is required to safeguard its vital cellular functions. Its deregulation is either induced directly by an altered gene expression, changed DNA methylation, abnormal mRNA regulation by for example lncRNAs or miRNAs, or indirectly by post-translational modifications *via* altered kinases such as Aurora A, Aurora B, Plk1 or Cdk1.

## 7. Study limitations and outlook

Although a lot of work has been done in recent years, most of the summarized data involved multi-omics approaches and computational science, which were heavily based on whole genomic transcriptomics. More detailed studies including proteomics are needed to solidify the significance of KIF2C as a diagnostic/prognostic marker for cancer patients, as clearly proposed for breast cancer, NSCLC and HCC (Figure 3(A–C)). Moreover, clinical trials including KIF2C expression data or the use of highlighted gene signatures for the design of treatment schedules for multiple cancer entities would largely expand the significance of KIF2C’s role in the outcome of patients. In addition, it is of importance to investigate whether the increase of KIF2C with its oncogenic potential is a driving factor in carcinogenesis because of its role in chromosome instability, or whether it is a pathological consequence of the unrestricted cell cycle of cancer cells.

Recent progress in establishing patient-derived tumor organoids (PDO) and stroma organoids in combination with CRISPR technologies provide novel tools to study the molecular regulatory network of KIF2C deregulation in elaborated *in vivo* models [205,206]. Moreover, PDOs enable us to recapitulate KIF2C’s involvement in therapy resistance toward known MT-interfering agents such as taxanes. It would be of great interest to find out if PDOs from taxane resistant patients have deregulated MT depolymerization machinery, compared to sensitive patients. Furthermore, the advances in conditional knockout mice confer the possibility to study KIF2C’s role during malignant tumor as well as organ formation and development, as shown recently for the nervous system in conditional knockout KIF2C^flox^/^flox^/Nestin^Cre^ mice [207]. These models can be combined with specific inhibitory small molecule compounds against KIF2C [89] or activating/inhibiting modifications on KIF2C introduced by the precise CRISPR technology, which make it possible to explore the individual roles of KIF2C during these vital cellular processes. The novel omics and scRNA-seq. technologies [208] will help to decipher in which cell population KIF2C is enriched within the TME and which pathways are involved in this process, as displayed by recent studies for KIF2C in PDAC [95,99]. Finally, it will be of great interest, if KIF2C is indeed able to modulate the immune response in the TME as suggested in some cancer entities and how this is facilitated by a protein mainly known for its MT destabilizing function. These modern tools and techniques will help to confirm that KIF2C can serve as a diagnostic and prognostic marker for patients with various cancers.

## Supplementary Material

Supplement Table 1

Supplement Figure 1

## Figures and Tables

**Table 1.** Correlation between KIF2C/MCAK expression and the clinicopathologic features of various cancer entities.

Tumor entities	Clinicopathologic features associated with KIF2C/MCAK overexpression	Reference
Gastric cancer	OS: OR 1.95 CI (1.21–3.36) *p* = 0.008)	[113]
	MLN: OR 4.18, 95% CI (1.87–17.86) *p* = 0.001	
	SI: OR 1.54, 95% CI (1.02–2.33) *p* = 0.039	
	OS: HR = 0.59 [0.41–0.83] *p* = 0.003	[177]
	DSS: HR = 0.58 [0.37–0.90] *p* = 0.015	
	PFI: HR = 0.52 [0.36–0.76] *p* = 0.001	
Colorectal cancer	KI-67 expression: *r* = 0.69; *p* = 0.0003	[115]
	MLN: *p* = 0.0023	[114]
	VI: *p* = 0.019	
	PI: *p* = 0.021	
	Duke’ classification: *p* = 0.0023	
	OS as gene signature with PARPBP, KNSTRN, and KIF2C: *p* = 0.0038	[187]
Breast Cancer	OS: HR = 3.77 [2.61–5.59] *p* = 0.0001 (GEO dataset)	[117]
	OS: HR = 2.55 [2.03–3.22] *p* = 0.0001 (TCGA dataset)	
	DFS: HR = 2.49 [1.58–4.18] *p* = 0.0001 (GEO dataset)	
	DFS: HR = 2.66 [1.93–3.66] *p* = 0.0001 (TCGA dataset)	
	Basal-like BC:	
	OS: HR = 1.61 [1.01–2.69] *p* = 0.0508 (GEO dataset)	
	DFS: HR = 1.62 [1.08–2.53] *p* = 0.0248 (TCGA dataset)	
	OS: HR = 2.65 [1.28–5.49] p = 2e^−03^	[123]
	NKI295 dataset:	[122]
	OS: *p* = 0.0001, DMSF: *p* = 0.0004	
	GIS251 dataset:	
	OS: *p* = 0.0224, RFS: *p* = 0.0042	
	OS: *p* = 0.0088	[127]
	Stage: *p* = 0.001	
	Tumor size: *p* = 0.001	
	OS: HR = 1.65 [1.07–2.53] *p* = 0.022 (http://kmplot.com/)	[132]
	DMFS: HR = 1.87 [1.53–2.27] *p* = 0.001 (http://kmplot.com/)	[131]
	stage: F-value = 5.20 (Pr(>F) = 0.0229	
	OS: HR = 1.63 [1.46–1.82] p = 1e^−16^ (http://kmplot.com/)	[134]
	OS: HR = 1.69 [1.39–2.04] *p* = 5.8e^−8^ (http://kmplot.com/)	[136]
	CD4^+^ T-cell: rho = 0.599, *p* = 2.2e^−16^	
	OS: HR = 1.81 [1.28–2.55] adjusted *p* = 0.001	[129]
	OS: HR = 1.69 [1.51–1.89] p = 1e^−16^ (http://kmplot.com/)	[126]
	OS: HR = 1.74 [1.40–2.16] p = 4.3e^−7^ (http://kmplot.com/)	[128]
	OS: *p* = 0.0001, grade: *p* = 0.05 (grade 2 vs grade 3)	[209]
	OS: *p* > 0.0001, RFS: *p* > 0.0001, DMFS: *p* > 0.0001	[135]
	OS: *p* = 2.72e^−2^ (TCGA dataset)	[130]
	OS: p = 9.02e^−3^ (GSE42568 dataset)	
	OS: *p* = 3.83e^−2^ (GSE20685 dataset)	
	OS: HR = 1.57 [1.08–2.27] *p* = 0.018 (univariate cox reg.)	
	OS: HR = 1.60 [1.05–2.43] *p* = 0.027 (multivariate cox reg.)	
	OS: HR = 1.69 [1.39–2.04] *p* = 5.8e^−08^ (http://kmplot.com/)	[138]
	RFS: HR = 1.66 [1.50–1.84] *p* = 1.0e^−16^ (http://kmplot.com/)	
	TNM staging: *p* ≤ 0.05	
	CD4+ T cell infiltration: *p* ≤ 0.05	
	CD8+ T cell infiltration: *p* ≤ 0.05	
Non-small cell lung cancer (NSCLC)	OS: HR =1.78 [1.57–2.03]	[161]
	TNM staging: *F-*value = 5.38 (Pr(>F) = 0.00119 (GEPIA)	
	OS: HR =1.98 [1.48–2.65] *p* = 2.5e^−06^ (GSE30219)	[163]
	OS: HR =3.88 [1.76–8.55] p = 3e^−04^ (GSE31210)	
	OS: HR = 1.82 [1.14–2.9] *p* = 0.011 (GSE50081)	
	OS: HR = 1.57 [1.22–2.02] *p* = 0.00037 (CaArray)	
	TNM staging: *p* = 0.0049	
	Differentiation: *p* = 0.0078	
	MLN: *p* = 0.0001	
	OS: HR = 1.4 [n.s.] *p* = 0.017 (GEPIA)	[162]
	TNM staging: F-value = 5.38 (Pr(>F) = 0.00119 (GEPIA)	
	OS: HR = 1.6 [n.s.] *p* = 0.0044 (GEPIA)	[168]
	OS: HR =2.58 [2.16–3.09] p = 1e^−16^ (http://kmplot.com/)	[169]
	OS: *p* = 0.015 (lung adenocarcinoma)	[165]
	OS: *p* = 0.020 (lung squamous cell carcinoma)	
	OS: HR = 2.18 [1.48–3.22] *p* = 5.2e^−05^ (http://kmplot.com/)	[164]
	OS: HR = 1.4 *p* = 0.017 (GEPIA dataset)	
	CD4+ T cell infiltration: *p* = 2.46e^−2^ (TIME dataset)	
	CD8+ T cell infiltration: *p* = 8.01e^−9^ (EPIC dataset)	
Hepatocellular carcinoma (HCC)	OS: *p* = 0.005, RFS: *p* = 0.041,	[142]
	Grade: *p* = 0.002, TNM: *p* = 0.013	
	survival outcomes: HR = 1.832 [1.06–2.51] *p* = 0.027	
	OS: univariate analysis HR = 1.053 [1.01–1.03] *p* = 0.0001	[149]
	OS: multivariate analysis HR = 1.043 [1.01–1.08] *p* = 0.0171	
	OS: HR = 2.96 [1.31–6.71] *p* = 0.0063 (http://kmplot.com/)	[150]
	RFS: HR = 2.13 [1.15–3.94] *p* = 0.014 (http://kmplot.com/)	
	OS: HR = 2.16 [1.51–3.08] *p* < 0.001	[151]
	PFI: HR = 2.01	
	[1.50–2.70] *p* < 0.001	
	patient collective:	[144]
	OS: HR = 4.00 [1.65–9.74] *p* = 0.002	
	DFS: HR = 1.76 [1.09–2.85] *p* = 0.021	
	Differentiation: *p* = 0.016	
	Relapse: *p* = 0.025	
	TCGA collective:	
	OS: HR = 2.13 [1.49–3.04] *p* = 0.003	
	DFS: HR = 1.77 [1.31–2.39] *p* = 0.021	
	OS: HR = 2.2 [n.s.] *p* = 1.1e^−05^	[141]
	DFS: HR = 1.8 [n.s.] p = 6.2e^−05^	
	NTN: *p* = 0.015	
	Tumor size: *p* = 0.009	
	OS: *p* = 0.012	[152]
	OS: HR = 1.31, *z* = 4.71, *p* = 2.49e^−06^ (GEPIA)	[153]
	HR = 2.2, *p* = 0.0012 (http://kmplot.com/)	
	DFS: HR = 1.8 p = 7.5e^−05^	
	Grading: *p* > 0.0001	
	AFPE: *p* = 0.001	
	OS: HR = 2.2, *p* = 1.8e^−05^ (GEPIA)	[98]
	OS: HR = 2.13 [1.49–3.04] *p* = 2.3e^−05^ (http://kmplot.com/)	
	DFS: HR = 1.9, *p* = 5.9e^−05^ (GEPIA)	
	RFS: HR = 1.77 [1.27–2.47] *p* = 0.0006 (http://kmplot.com/)	
	Staging: F-value = 11.1 (Pr(>F) = 5.59e^−07^	
	OS: HR = 1.2 [0.99–1.50] *p* = 0.05	[145]
	OS: HR = 2.29 [1.69–3.10] *p* < 0.001 (ICGC)	[154]
	OS: *p* = 1.1e^−05^ (GEPIA)	
	OS: HR = 1.47 [1.22–1.77] *p* < 0.001 (TCGA)	
	OS: HR = 2.2, *p* = 1.7e^−05^	[102]
	Immune cell infiltration: *p* < 0.001	
Glioma	OS: *p* < 0.0001	[174]
	OS: HR = 4.73 [1.79–12.45] *p* < 0.001	[173]
	OS: HR = 2.15 [1.26–3.65] *p* = 0.005	[103]
	Grading: HR = 3.40 [2.30–5.02] *p* < 0.0001	
	Radio therapy: HR = 2.05 [1.19–3.54] *p* = 0.01	
	Grading: *p* < 0.005	[172]
	OS: WHO Grade I–II; *p* = 0.026	
	OS: WHO Grade III; *p* = 0.043	
	OS: WHO Grade IV; *p* = 0.059	
Endometrial cancer (EC)	OS: HR = 1.4 [0.97–1.9] *p* = 0.019	[189]
	OS: *p* = 0.0097	[191]
	Differentiation: HR = 1.23 [1.03–1.42] *p* = 5.45e^−35^	
	OS: *p* = 0.00537	[190]
	OS: *p* = 0.012	[192]
	Staging: *p* < 0.01	
	Grading: *p* < 0.01	
	Histological type: *p* < 0.01	
Ovarian cancer (OC)	OS: KIF2C low expression – HR = 1.15 [1.01–1.32] *p* = 0.038 (http://kmplot.com/)	[193]
	OS: HR = 0.94 [n.s.] *p* = 0.59	[197]
	Staging: F-value = 2.92 (Pr(>F) = 0.0548	
	OS: KIF2C low expression – HR = 1.15 [1.01–1.32] *p* = 0.038 (http://kmplot.com/)	[196]
Esophageal squamous cell carcinoma (ESCC)	OS: HR = 1.48 [1.085–2.020] *p* = 0.013 (male)	[176]
	DFS: HR = 1.42 [1.048–1.918] *p* = 0.024 (male)	
	OS: *p* = 0.880 (female)	
	DFS: *p* = 0.864 (female)	
	Differentiation: *p* = 0.022	
	pT status: *p* = 0.038	
	OS: HR = 0.37 [0.13–1.04] *p* = 0.05	[210]
Kidney renal clear cell carcinoma (KIRC)	OS: HR = 1.5 [n.s.] *p* = 0.0057	[95]
	DFS: HR = 1.8 [n.s.] *p* = 0.0018	
	OS: HR = 2.14 [1.756–2613] *p* < 0.001	[105]
	Staging: *p* < 0.001	[184]
	OS: HR = 1.5, pHR = 0.006, *p* = 0.0057	
	DFS: *p* = 0.0018	
	OS: *p* < 0.001	[118]
Pancreatic Ductal Adenocarcinoma (PDAC) & Pancreatic Adenocarcinoma (PAAD)	OS: HR = 1.6, pHR = 0.021, *p* = 0.02 (GEPIA dataset)	[99]
	DFS: HR = 2.2, pHR = 0.00072, p = 5e^−4^	
	PFS: *p* = 0.007	
	PST: *p* = 0.0033	
	OS: HR = 1.6, pHR = 0.021, *p* = 0.02 (GEPIA dataset)	[179]
	OS: HR = 1.4 [1.04–1.9] *p* = 0.026 (TCGA-PAAD)	
	Grading: *p* = 0.001	
	Tumor growth: *R* = 0.73, p < 2.2e^−16^	
	OS: HR = 1.6, pHR = 0.021, *p* = 0.02 (GEPIA dataset)	[180]
	DFS: HR = 2.2, pHR = 0.00072, p = 5e^−4^	
	OS: *p* = 0.057 (GSE 62452 dataset)	
	OS: HR = 1.42 [1.177–1.723] *p* < 0.001	[181]
	DFI: HR = 1.54 [1.087–2.183] *p* = 0.015	
	DSS: HR = 1.48 [1.193–1.844] *p* < 0.001	
	PFI: HR = 1.41 [1.179–1.684] *p* < 0.001	

Abbreviations: MLN: metastatic lymph node; SI: serosal invasion; OR: odds ratio; VI: venous invasion; PFI: progression free interval; PI: peritoneal dissemination; PST: postoperative survival time; PAAD: pancreatic adenocarcinoma; DFS: disease-free survival; DMSF: distant metastasis free survival; DSS: disease specific survival; RFS: relapse free survival; TCGA: the cancer genome atlas; NTN: number of tumor nodes; AFPE: α-fetoprotein expression; pT: pathologic tumor.

* All values show the significance of high KIF2C expression on the malignant features of the cancer entities.

**Table 2.** Molecular mechanisms involved in KIF2Cs oncogenic potential in multiple cancer entities/cell lines.

Tumor Entity	Experimental Setup	Molecular mechanism and main results	Reference
Breast cancer (BC)	• Breast cancer tissue, KIF2C RT-PCR, *n* = 14	*KIF2C* is overexpressed in 12 of 14 clinical breast cancer cases.	[121]
	• *In vitro*: cell lines T47D and HBC5, knockdown by siRNA	Knockdown of MCAK inhibited cell growth.	
	• *In vitro*: breast cancer cell line MDA-MB-231, siRNA	In absence of *KIF2C*: lower migration and invasion capacity with reduced paxillin, p-paxillin, FAK, and p-FAK intensity in focal adhesions (FAs), and a decreased FA size	[36]
	• *In silico*: public databases concerning expression profile of miR-10b-5p in breast cancer (METABRIC, TCGA, GSE40267 and GSE19783)	*KIF2C* is a key target of miR-10b-5p. *KIF2C* was mainly involved in sustaining proliferative signaling in cancer development.	[116]
	• *In vitro*: breast cancer cell line MDA-MB-231, miR-10b-inhibitor, RT-PCR	Inhibitor treatment enhanced *KIF2C* gene expression (n.s.).	
	• *In silico*: large-scale gene expression datasets of breast cancer (total *n* = 4,677) and microRNAs targeting MCAK predicted by bioinformatic analysis (TCGA, GSE7390, GSE2034, GSE1456, GSE4922, GSE22226, GSE24450, GSE53031, GSE25066, GSE10885, GSE58812, and NKI)	*KIF2C* expression was significantly associated with aggressive features of breast cancer and associated with poor outcome in a dose-dependent manner for either ER-positive or ER-negative breast cancer. miR-485–5p and miR-181c might target *KIF2C* and suppress its expression, associated with better outcome.	[117]
	• *In vitro*: validated by a dual-luciferase reporter assay in breast cancer cell lines MCF-7 and MDA-MB-231		
	• *In vitro*: 39 FFPE and fine needle aspiration biopsies (FNAB) of 25 breast cancers of different clinical subtypes (based on ER and Her2/neu status); RNA-seq. and RT-PCR	Overexpression of *ANLN* and *KIF2C*, and lower expression of *MAPT* strongly correlated with poor outcomes in breast cancer patients.	[122]
	• *In silico*: public database BioGRID and six GEO datasets (GSE70947, GSE15852, GSE20711, GSE65212, GSE18229-GPL887, GSE65194)	Four genes, *FAM134B*, *KIF2C*, *ALCAM, KIF1A*, were identified having comparable subtyping efficiency with the initial 1015 breast cancer subtyping genes. *KIF2C* and *KIFC1* had a negative effect on patient clinical outcome.	[126]
	• *In silico*: GEO database: GSE10780 with 42 normal breast tissues and 143 IDC (invasive ductal carcinoma) tissues	Top 10 significantly up-regulated hub genes, *CDK1*, *CCNB1*, *CENPE*, *CENPA*, *PLK1*, *CDC20*, *MAD2L1*, *HIST1H2BK*, *KIF2C* and *CCNA2*	[127]
		*KIF2C*, *MAD2L1* and *PLK1* were associated with decreased overall survival (OS).	
	• *In silico*: GEO database: GSE25066 and GSE21422; basal-like breast cancer (BLBC)	Top 10 hub genes: *CCNB2*, *BUB1*, *NDC80*, *CENPE*, *KIF2C*, *TOP2A*, *MELK*, *TPX2*, *CKS2* and *KIF20A*	[128]
		Four hub genes, *BUB1*, *CCNB2*, *KIF2C* and *CDCA8*, were confirmed to be enhanced in basal subtype by RT-PCR.	
	• *In silico*: TCGA with 1055 BC patients	High levels of *KIF15*, *KIF20A*, *KIF23*, *KIF2C* and *KIF4A* genes were significantly correlated with poor OS. BC patients with high expression of *KIF15*, *KIF20A*, *KIF23*, *KIF2C* and *KIF4A* were enriched in the cell cycle process, p53 regulation pathway and mismatch repair.	[129]
	• *In silico*: TIMER, TCGA, KM-plotter, GSE36295, GSE42568, GSE20685	*KIF2C* is upregulated in 18 different types of cancer, including breast cancer. *KIF2C*^*high*^ expression was correlated with poor OS, TMN staging, T status and molecular subtypes. *KIF2C* was associated with immune cell infiltration, tumor mutation burden and response to immunotherapy.	[130]
	• *In silico*: GSE27447, GSE43358, GSE36295, GSE61724, GSE75678, TCGA	Top 5 hub genes are associated with triple negative breast cancer: *TPX2, CTPS1, KIF2C, MELK* and *CDCA8.*	[131]
		Highest deregulated pathways: cell cycle, oocyte meiosis and spliceosome.	
	• *In silico*: KM-plotter, TCGA, Oncomine, GEPIA, GSE81540	The following genes were found to be important for stemness maintenance of TNBC: *BIRC5, CDC25A, KIF18B, KIF2C, ORC1, RAD54L* and *TPX2.*	[132]
		A high expression of *BIRC5, KIF2C, KIF18B,* and *TPX2* reduced RFS and free survival.	
	• *In silico*: KM-plotter, GSE102484, GSE102484, TCGA	Five hub genes for breast cancer were identified: *TPX2*, *KIF2C*, *CDCA8*, *BUB1B*, and *CCNA2*. The expression of *KIF2C* correlated with higher breast cancer staging, had a diagnostic value in identifying normal and breast cancer tissue and had prognostic value for DMFS.	[133]
	• *In silico*: GSE109169, GSE115144, TCGA, GEPIA, cBioPortal, TISIDB, E-MTAB-365, GSE11121, GSE12093, GSE12276, GSE1456, GSE16391, GSE16446, GSE16716, GSE17705, GSE17907, GSE19615, GSE20271, GSE2034, GSE20685, GSE20711, GSE21653, GSE2603, GSE26971, GSE2990, GSE31519, GSE3494, GSE37946, GSE42568, GSE45255, GSE4611, GSE4922, GSE5327, GSE6532, GSE7390, GSE9195	*KIF2C* was found to be a hub gene among 9 other genes. The overexpression of these genes was verified in breast cancer tissue and all of these hub genes were associated with a negative overall survival in breast cancer patients. The expression levels of *CDK1*, *CENPF*, *KIF2C*, *KIF4A*, *MELK*, *PBK*, *PRC1*, and *TPX2* were associated with increased CD4^+^ T cell infiltration.	[136]
	• *In silico*: GSE29044, GSE42568, GSE50428, TCGA, KM-plotter	Twelve hub genes were found, including *KIF2C*, and the expression of *KIF2C* was associated with reduced OS.	[134]
	• *In silico*: TCGA, GTEx project, GEPIA 2, KM-plotter, METABRIC	This study found 20 different expressed genes between normal tissue and breast cancer tissue. Among these 20 genes, *KIF2C* was highly overexpressed and correlated with poor OS, RFS and DMFS.	[135]
	• *In silico*: TCGA, GEPIA2, HPA, ROC, KM-plotter	Thirteen hub genes were found: *CDK1*, *BUB1*, *BUB1B*, *CDC20*, *CCNB2*, *CCNB1*, *KIF2C*, *NDC80*, *CDCA8*, CENPF, *BIRC5*, *AURKB*, *PLK1*, *MAD2L1*, and *CENPE*. The performance as prediction marker was corroborated by a receiver operating characteristic analysis and found that the area under the curve value for *KIF2C* was 97.6%, highlighting its prognostic potential.	[140]
	• *In silico*: TCGA, KM-plotter, GSE47561, GSE25066	*KIF2C* was significantly overexpressed in breast cancer tissue. This expression was the highest in triple negative breast cancers. KIF2C^high^ expression correlated with reduced OS and DMSF. Knockdown of KIF2C significantly enhances the paclitaxel sensitivity of breast cancer cells, without affecting normal cells. In a drug-discovery approach they found a potent KIF2C inhibitor, which induced paclitaxel sensitization and reduced the clonogenic survival of the triple negative breast cancer cell line MDA-MB-231.	[137]
	• *In vitro* (cell lines): MDA-MB-231, PTXR231, MCF10A, and E8.1 HeLa		
	• *In vitro:* MT depolymerization assay was used to verify potential KIF2C inhibitors		
	• *In silico*: GSE10797, GSE15852, GSE92697, GSE102484, GSE65212, GSE43837, GSE23988, GSE20194, GSE42568, GSE75333, GSE5847, GSE22035, GSE3744, GSE5764, GSE21422, GSE26910, GSE41970, GSE8977, GSE45827, GSE71142, GSE86945, GSE86946, GSE29431, GSE65194, GSE22093, GSE31192, GSE9014, GSE10780, GSE29431, GSE61304, GSE10810 and KM-plotter	Six hub genes were found in the comprehensive breast invasive carcinoma samples including Centrosomal protein of 55 kDa (*CEP55*), *KIF2C*, *KIF20A*, Ribonucleotide Reductase Regulatory Subunit M2 (*RRM2*), *AURKA*, and Protein Regulator of cytokinesis 1 (*PRC1*). These were verified by expression profiling and validation analyses, Further, these genes were associated with altered promoter methylation status, genetic alteration, reduced overall survival (OS), relapse-free survival (RFS), tumor purity, CD8+ T, CD4+ T immune cell infiltration, and different mutant genes across BRIC samples.	[138]
Hepatocellular carcinoma (HCC)	• *In silico*: TCGA database and tissue: 66 HCC surgical specimens, IHC	High KIF2C expression in HCC. KIF2C expression was associated with HCC prognosis, including OS and DFS. KIF2C expression was also associated with clinical pathological characteristics including the number of tumor nodes and tumor size.	[141]
	• *In vitro*: HCC cell lines Hep3B and SNU475 treated with shRNA against KIF2C		
	• *In vivo*: xenograft mouse model with control or KIF2C stably depleted Hep3B cells	KIF2C knockdown inhibited proliferation of HCC cells *in vitro* and suppressed tumor growth in mice.	
	• *In silico*: TCGA with 295 HCC patients	Expression of 32 KIF genes was studied. *KIF2C*, *KIF4A*, and *KIF11* overexpression were significantly associated with shorter relapse-free survival times. *KIF2A*, *KIF2C*, *KIF3A*, *KIF4B*, *KIF11*, *KIF15*, *KIFC*1, and *KIFC3* overexpression was associated with shorter OS times. *KIF2A*, *KIF2C*, *KIF4A*, *KIF4B*, *KIF5B*, *KIF7*, *KIF11*, *KIF15*, *KIF24*, *KIF27*, and *KIFC1* were biomarkers for HCC progression	[142]
	• *In silico*: TCGA database with 424 tissue samples including 374 HCC and 50 normal samples, merged with the corresponding mRNA expression-based stemness index (mRNAsi)	44 highly-correlated significant elevated genes were screened and functionally related to cell cycle, cellular senescence, p53 signaling pathway, DNA replication, and mismatch repair. 15 key biomarkers, i.a. *KIF4A* and *KIF2C*.	[143]
	• *In silico*: TCGA, Oncomine data, a transcriptome study of 225 primary HCC and 220 normal liver tissues, verified with 39 and 12 paired HCC and adjacent tissues (RT-PCR and WB) and IHC from 149 patients with HCC	KIF2C expression was increased in almost all cancer types, including HCC.	[144]
		KIF2C was significantly overexpressed in HCC compared to normal liver tissues.	
		KIF2C upregulation is frequently observed in HCC and correlates with a poor prognosis.	
	• *In vitro*: gain and loss of function assays: HCC cell lines SK-Hep1 and SNU449 with shRNA, and HepG2 and SNU387 cells with overexpressed KIF2C	KIF2C promoted HCC cell proliferation, migration, invasion, and metastasis both *in vitro* and *in vivo*. TBC1D7 was identified as a binding partner of KIF2C, this interaction disrupts the formation of the TSC complex, causing enhancement of mammalian target of rapamycin complex1 (mToRC1) signal transduction. KIF2C is a direct target of the Wnt/β-catenin pathway, establishing a link between Wnt/β-catenin and mToRC1 signaling, highlighting KIF2C as a potential target for the treatment of HCC.	
	• *In vivo*: xenograft nude mice experiments with HepG2-KIF2C cells and KIF2C-depleted SK-Hep1 cells		
	• KIF2C IHC (20 HCC and adjacent non-tumor liver tissues) and RT-PCR (76 patients, including cancerous and matched non-cancerous tissues) from tissue samples, verified by GEO (GSE14520 dataset), TCGA and Oncomine database included 225 HCC and 220 normal cases	KIF2C is overexpressed in HCC, related to neoplasm histologic grade, pathology stage, and a dismal prognosis (overall, recurrence-free, and disease-free survival). The diagnostic efficacy of KIF2C was >90% in HCC.	[98]
	• *In vitro*: HCC cell lines Hep3B and Huh7, overexpression with lentiviral vectors and downregulation with shRNA; RNA-seq.	Cell culture: KIF2C promoted cell proliferation, migration and invasion through EMT, and an accelerated cell cycle, and inhibited apoptosis. KIF2C promoted HCC through the Ras/MAPK and PI3K/Akt signaling pathway.	
	• TCGA database	Increased KIF2C expression in HCC associated with poor prognosis.	[145]
	• *In vitro*: Hep-3B, MHCC97H, HCCLM3, Huh7, and an immortalized normal liver epithelial cell line (THLE-3), KIF2C siRNA	Silencing KIF2C significantly suppressed proliferation, migration, and invasion in HCC cells with decreased expression of Snail, Vimentin, p-MEK, and p-ERK, but increased E-cadherin. The MEK/ERK inhibitor U0126 enhanced the impact on cell proliferation, migration, and invasion induced by silencing, the MEK/ERK activator PAF.	
	• *In vitro*: RNA-seq. of Huh-7-control and ANLN-KD cells, RT-PCR confirmation; ectopic KIF2C expression	HCC BM was associated with enhanced nuclear ANLN, which formed a transcriptional complex with SP1, which enhanced KIF2C transcriptional activity to activate the mToRC1 pathway. KIF2C significantly reduced osteoclast differentiation activity promoted by ectopic ANLN expression. Decreased phosphorylation of AKT/increased β-catenin observed in ANLN-KD HCC cells could be partially recovered by ectopic KIF2C expression.	[147]
	• *In silico*: various databases, i.a. ONCOMINE database, TCGA, human protein atlas; verification: cancer tissues and normal liver tissues of 15 patients with liver cancer for IHC	Kinesin family members KIF2C/4A/10/11/14/18B/20A/23 predict poor prognosis (significantly associated with the tumor stage and pathological tumor grade; shorter OS and DFS). Cox regression analysis showed the mRNA expression levels of these KIFs is an independent prognostic factor for worse outcomes in HCC. KEGG: the cell cycle, p53 signaling pathway, PPAR signaling pathway and DNA replication	[153]
	• *In vitro*: HCC cell lines SK-Hep-1, Huh7, and HepG2. The HCC cell lines BEL-7404 and HCC-LM3 and the normal human liver epithelial cell line HL-7702; siRNA	*In vitro*: downregulation of KIF proteins effectively decreased the proliferation and colony formation, increased G1 arrest.	
	• *In silico*: TCGA, Human-GEM, BRENDA, GSE14520	The study found two genes with independent prognostic value: *KIF2C* and *ATIC*. The overexpression of both genes was associated with poor OS.	[149]
	• *In silico*: GSE47197, GSE55092, GSE121248	*KIF2C* was found within the highest deregulated genes in the HCC cohort. Its expression was verified in HBV-related HCC and *KIF2C*^*high*^ expression was correlated with poor OS and decreased RFS.	[150]
	• *In silico*: GSE17548, TCGA GSE25097, KM-plotter	Six hub genes were identified: *BUB1B*, *NUSAP1*, *TTK*, *HMMR*, *CCNA2*, and *KIF2C*. All of these genes had a high diagnostic value for HCC with reduced OS, PFI and an altered immune cell infiltration.	[151]
	• *In silico*: GSE62322, GSE112790, GSE102079, GSE14323, GSE14520, GSE89377, GSE64041, TCGA	Fourteen hub genes were identified including *KIF2C*. These genes were significantly upregulated in HCC and associated with a decreased OS. *KIF2C* was also correlated with an increased infiltration of regulatory T cells (Treg), T follicular helper (TFH) cells, macrophages M_0_, but negatively correlated with the infiltration of monocytes.	[152]
	• *In silico*: GSE14520, GSE22058, ICGC, TCGA	A network between 10 genes including *KIF2C* was found associated with clinicopathological features (reduced OS) of HCC. All of these genes are independent predictors of HCC and were connected to immune cell infiltration.	[154]
	• *In silico*: TCGA, ICGC, TCIA	This study generated a hallmark-guided subtypes-based immunologic signature (HGSIS) including eight genes. *KIF2C* was calculated as a key target concerning this HGSIS and ten small molecules were predicted to bind to the active site of KIF2C *via* molecular docking analysis, which might be useful for future therapies.	[155]
	• *In silico*: TCGA-LIHC, GSE14520	This study generated cuproptosis-linked subtypes and found a prognosis risk model related to cuproptosis with seven key genes: *KIF2C*, *PTTG1*, *CENPM*, *CDC20*, *CYP2C9*, *SFN*, and *CFHR3*. This HCC subtype had a reduced survival prediction.	[157]
	• *In silico*: TCGA-LIHC, GSE14520, HPA	An endoplasmic reticulum-related prognostic signature for HCC was generated included KIF2C. The generated signature was associated with reduced OS and RFS. Additionally, it was connected to increased macrophages M_2_/M_0_ and regulatory T cells infiltration.	[159]
	• *In silico*: GEO (normal samples-PRJNA494560, tumor samples-PRJNA414787), KM-plotter	Identification of thirteen hub genes for HCC and glioblastoma multiforme including *KIF2C. KIF2C* was overexpressed in both cancer entities, hypomethylated and *KIF2C*^*high*^ expression was correlated with poor OS.	[160]
	• *In silico*: TCGA (included 374 HCC tissue samples and 50 adjacent non-tumor samples), ICGC, WGCNA, IMvigor210	A novel prognostic signature for HCC was established using the protein and gene expression profiles of KIF2C and RAC1. Patients with high expression of both genes were assigned to a “high-risk group” with significant worse prognosis, higher clinicopathological grade, increased levels of tumor mutation burden, enhanced CD8+ T cell infiltration, increased expression of immune checkpoint genes, enhanced immunotherapy efficacy and an altered sensitivity toward chemotherapy drugs.	[156]
Non-small cell lung cancer (NSCLC)	• *In silico*: GSE18842 (included 46 NSCLC tissue samples and 45 paired non-tumor samples), GSE21933 (21 NSCLC tissue samples and 21 corresponding normal samples), and GSE89039 (eight LAC tissues samples and eight controls); TCGA	The hub genes, including *AURKB*, *BUB1B*, *KIF2C*, *HMMR*, *CENPF*, and *CENPU*, were overexpressed compared with the normal group and associated with worse OS and TNM stage.	[161]
		Genomic alterations of KIF2C: 2.1%	
	• *In silico:* GSE31210 containing LUAD (*n* = 226) and normal lung tissue (*n* = 20)	High expression of *KIF2C* is related to the relapse and tumor stage of LUAD and reduced OS.	[162]
	• NSCLC tissues and normal lung tissues; 100 patients	KIF2C is up-regulated in NSCLC tissues and cell lines. High expression level of KIF2C predicted worse survival.	[163]
	• *In vitro*: NSCLC cell lines A549, H1299, H226, and H520. A549 and H226 were transfected with shRNA	KIF2C knockdown inhibited proliferation, colony formation, migration and invasion of NSCLC cell lines. *KIF2C* was a target gene of miR-325-3p, which was reported to be a tumor suppressor in NSCLC.	
	• *In silico:* GEO dataset (GSE18842, GSE32863, GSE75037, GSE81089)	key genes involved in the process of lung cancer: *AURKA*, *CCNB1*, *KIF11*, *CCNA2*, *TOP2A*, *CENPF*, *KIF2C*, *TPX2*, *HMMR*, and *MAD2L1*	[167]
	• *In silico*: microarray datasets GSE18842 and GSE118370	Protein protein interaction network analysis revealed several hub genes: CDK1, CDC20, *BUB1*, *BUB1B*, *TOP2A*, *CCNA2*, *KIF20A*, *CCNB1*, *KIF2C*, and *NUSAP1*.	[168]
	GSE18842: 91 samples (45 control samples and 46 NSCLC samples) and GSE118370: 12 samples (6 controls and 6 NSCLC samples)	High *KIF2C* expression associated with reduced OS.	
	• *In silico:* GSE37745 including a total of 196 NSCLC samples; CIBERSORT package	High expression of *AURKB*, *CDC20*, *TPX2*, and *KIF2C* correlated with poor prognosis. *AURKB*, *CDC20*, *TPX2*, and *KIF2C* are potential CD8+ T cell infiltration-related biomarkers and therapeutic targets.	[169]
	• In silico: GSE74706 and GSE149507	9 genes (*AURKA*, *AURKB*, *KIF23*, *RACGAP1*, *KIF2C*, *KIF20A*, *CENPE*, *TPX2* and *PRC1*) were overexpressed in LC patients, potential prognostic biomarkers. overexpression leads to increased cell number and proliferation, potentially leading to cancer formation.	[170]
	The NSCLC and SCLC datasets comprised 36 samples each with 18 normal and 18 tumor paired samples	Frequency of mutations: 1.5%.	
	• *In silico*: collectively analyzing 31 GEO expression datasets of lung adenocarcinoma (LUAD), KIF2C included in GSE68465;	Upregulated hub genes: *CDC6*, *PBK*, *AURKA*, *KIF2C*, *OIP5*, and *PRC1*, associated with poor OS	[164]
	UALCAN/TCGA (consisting of 515 cancerous and 59 normal samples); GENT2, GEPIA, DriverDBV2, ENCORI	KIF2C is significantly hypomethylated relative to controls in LUAD patients. KIF2C had a genomic alteration rate of 3%. CD8+ T, and CD4+ T immune cells infiltration were negatively correlated with KIF2C.	
		E2F1 and hsa-mir-34a-5p targeted all hub genes and were significantly upregulated in LUAD.	
	• *In silico:* TCGA	High KIF2C expression in LUAD and LUSC tissues were associated with worse prognosis.	[165]
	• *In vitro*: normal lung epithelial cell line BEAS-2B and lung cancer cell lines, including A549, A549-DDP, LLC, PC-9, SHP-77 and NCI-H1703; vector expression or siRNA	The results showed that KIF2C was upregulated in NSCLC cell lines. KIF2C overexpression promoted proliferation, migration, and invasion, and inhibited apoptosis.	
		KIF2C was as a key target of miR-186–3p. High expression of KIF2C increased the levels of β-catenin, p-GSK-3β and p-AKT. KIF2C downregulation and miR-186–3p upregulation reversed these results.	
Gastric cancer (GC)	• tissue: 65 cases of gastric cancer; RT-PCR and IHC	Elevated KIF2C expression was associated with lymphatic invasion and lymph node metastasis. Patients with high KIF2C expression had a significantly poorer survival rate.	[113]
	• *In vitro*: cell line AZ521 KIF2C vector-overexpression		
		Overexpression of KIF2C increased cell proliferation, migration and resistance against anoikis.	
	• *In silico:* GEPIA, TIMER	KIF2C was overexpressed and might be used as a diagnostic and prognostic biomarker. KIF2C expression was correlated with immune infiltration and cell cycle-related genes.	[177]
	• tissue samples: 35 GC samples and their corresponding normal samples	Knockdown of KIF2C caused cell cycle arrest and decreased the proliferation of GC cell lines. overexpression of KIF2C had the opposite effect.	
	• *In vitro*: cell lines SNU-1 knockdown by siRNA and AGS KIF2C-overexpression vector		
	• *In vitro*: cytotoxic T lymphocytes (CTLs) were primed with KIF2C peptides	KIF2C peptide primed CTLs were able to lyse various colon and NUGC3 gastric cancer cell lines in a peptide-based immunotherapeutic assay.	[178]
	• *In silico:* TCGA, GTEx database, GEPIA2, CCLE, UALCAN, CPTAC, HPA, TIMER2; verified with IHC of gastric cancer samples	KIF2C is significantly higher in tissue of gastric tumor patients.	[95]
	• *In vitro*: GES-1, SGC-7901, HGC-27, MKN-45, and AGS	KIF2C is highly expressed in gastric cancer cell lines, gastric adenocarcinoma, and hepatocellular carcinoma. KIF2C gene is highly expressed in SGC-7901, HGC-27, and MKN-45 when compared to GES-1 and AGS.	
Colorectal cancer (CC)	• cell lines: colorectal cancer cell lines HT29, COLO205, LIM1215, SKCO1, DLD1	KIF2C is significantly overexpressed and correlates with Ki-67 and the proliferative activity of CC. KIF2C was able to induce spontaneous CD4+ T cell responses of the Th1-type.	[115]
	• tissue: tumor samples and adjacent healthy tissues from 75 colorectal patients, RT-PCR+WB		
	• cell lines: DLD1, COLO201, COLO205, COLO320DM, HT29, HCT15, WiDr, SW480, LS174T, CCK81, RCM1 and CaR1, RT-PCR	KIF2C is expressed in various colorectal cancer cell lines, exception CoLo201.	[114]
	• tissue: paired colorectal tissue samples from tumors and the corresponding normal tissues were obtained from 120 patients, RT-PCR + IHC	KIF2C expression was higher in colorectal cancer tissue, and markedly associated with lymph node metastasis, venous invasion, peritoneal dissemination and Dukes’ classification. Patients with high *KIF2C* mRNA expression showed a poorer survival rate.	
	• *In silico*: gene expression profiles of 17 paired CRC and adjacent normal tissues (GSE110224) from the GEO database	Higher expression of the key genes (*KIF2C*, *CDC45*, *CEP55* and *DTL*) in CRC stages I–IV, adenocarcinoma and mucinous adenocarcinoma were identified as a stemness-related gene signature.	[188]
	• *In silico*: RNA-seq. data from 616 CRC patients from TCGA: 433 tissues and 408 colon adenocarcinoma (COAD), and 221 tissues and 208 rectum adenocarcinoma (READ)	The study identified 34 key genes as candidate prognosis biomarkers. A three-gene prognostic signature (*PARPBP*, *KNSTRN*, and *KIF2C*) was correlated with poor prognosis.	[187]
	• *In silico:* computational prediction of disease-associated non-synonymous polymorphism (nsSNP)	Mutation of E403K in KIF2C is associated with colorectal cancer interfering with protein conformation and stability.	[96]
Glioma	• tissue: 40 tumor samples and 6 normal brain tissues	*KIF2C*, *AURKB* and *Ki-67* genes were higher expressed in glioma samples and associated with histopathological grades.	[172]
	• *In vitro*: biomarker *KIF2C*, *AURKB* and *Ki-67* were tested by RT-PCR and KIF2C by WB in patient tumor samples	Multivariate survival analysis identified high expression of *KIF2C* and *Ki-67* as independent prognostic factors for survival time. *KIF2C* overexpression correlates with poor outcome. The expression level of *KIF2C* strongly correlated with proliferation marker *Ki-67*.	
	• *In silico*: TCGA; including 142 glioblastoma multiforme (GBM) patients	Top 10 hub genes (*CDC20*, *NCAPH*, *CDCA5*, *BUB1*, *CDCA8*, *PBK*, *KIF2C*, *TPX2*, *TTK* and *TOP2A*)	[173]
	• *In silico*: TCGA, 530 cases of low-grade glioma (LGG) and 173 cases of (GBM); GSE16011, 284 gliomas	10 hub genes (*CDC20*, *FN1*, *AURKB*, *AURKA*, *KIF2C*, *BIRC5*, *CCNB2*, *UBE2C*, *CCNA2*, and *CENPE*).	[174]
		*CDC20*, *FN1*, *AURKB*, *AURKA*, *KIF2C*, *BIRC5*, *CCNB2*, *UBE2C*, *CCNA2*, and *CENPE* may be potential biomarkers and therapeutic targets for LGG.	
	• *In silico*: CGGA database, 142 LGG and 34 sGBM samples	Five hub genes: *CCNB2*, *KIF2C*, *CDC20*, *TPX2*, and *PLK1* play an important role in promoting the transition of LGG to sGBM.	[175]
	• tissue: LGG, *n* = 8; sGBM, *n* = 4; IHC	High *KIF2C* expression is associated with poor OS.	
	• *In silico*: CGGA, TCGA; GSE63678 and GSE17025	*KIF2C* was identified as a hub gene in GBM with positive association between *KIF2C* expression and the advanced stages of gliomas. Higher expression of *KIF2C* in WHo grade IV samples relative to that in grade III and grade II samples. Higher *KIF2C* expression correlated with shorter survival time in both primary and recurrent gliomas. Higher *KIF2C* expression was related to higher levels of endothelial cell, T cell CD8+ naïve, common lymphoid progenitor, T cell CD4+ Th2, T cell CD4+ Th2, macrophage, macrophage M1, T cell CD4+ memory, and T cell CD4+ effector memory, but was related to lower levels of NK cell, B cell plasma, T cell CD4+ Th1, T cell regulatory (Tregs), neutrophil, and T cell NK.	[103]
	• *In silico*: GSEA for immune activity of glioma, TCGA database (from 508 glioma patients) and GEO (GSE4412, 85 glioma patients)	14 core genes were identified, namely: *TOP2A*, *TPX2*, *BUB1*, *AURKB*, *AURKA*, *CDK1*, *BUB1B*, *CCNA2*, *CCNB2*, *CDCA8*, *CDC20*, *KIF11*, *KIF20A*, and KIF2C.	[104]
Endometrial cancer (EC)	• *In silico*: GEO datasets (i.e. GSE63678, GSE17025) and TCGA	15 genes were associated with the prognosis of EC (*CCNB2*, *CDC20*, *BUB1B*, *UBE2C*, *AURKB*, *FOXM1*, *NCAPG*, *RRM2*, *TPX2*, *DLGAP5*, *CDCA8*, *CDC45*, *MKI67*, *BUB1*, *KIF2C*). The high expression was associated with poor prognosis.	[189]
	• *In silico:* TCGA of 552 primary tumor samples and 35 solid normal tissue samples	*AURKA*, *CENPA*, and *KIF2C*, were found to be potential biomarkers for EC with a significant prognostic effect.	[190]
	• *In silico*: TCGA including 548 patients with uterine corpus endometrial carcinoma (UCEC)	*AURKA*, *BUB1*, *CDCA8*, *DLGAP5*, *KIF2C*, and *TPX2* genes were significantly negatively related to the 5-year OS in patients.	[191]
	• *In silico*: TCGA (575 cases, including 23 normal cases)	KIF2C expression was higher in EC. Bioinformatics indicated that *KIF2C* is negatively correlated with the infiltration level of CD8+ T cells but positively with poor prognosis.	[192]
	• tissue: twelve EC tissues and twelve adjacent tissues; RT-PCR, WB + IHC	KIF2C knockdown prolonged G1 and inhibited EC cell proliferation, migration, and invasion *in vitro*. Apoptosis of CD8+ T cell was inhibited after KIF2C knockdown.	
	• *In vitro:* human EC cell lines Ishikawa and RL95–2; shRNA knockdown	KIF2C knockdown inhibited Ki-67 expression and tumor growth in a xenograft mouse model.	
	• mouse model with KIF2C-silenced Ishikawa cells		
Ovarian cancer (OC)	• GEO datasets (GSE6008, GSE18520, GSE26712, GSE27651, GSE29450, GSE36668, GSE52037) containing 396 ovarian cancer samples and 54 healthy control; differentially expressed genes (DEGs)	12 hub genes: *CDK1*, *TOP2A*, *CDC20*, *CCNB2*, *BIRC5*, *UBE2C*, *BUB1*, *NCAPG*, *RRM2*, *KIF2C*, *CENPA*, and *MELK* were analyzed.	[193]
		*KIF2C* gene expression is upregulated in OC and negatively associated with OS.	
	• *In silico*: GEO mRNA dataset (GSE28739, 30 chemo-resistant serous EOC samples and 20 chemo-sensitive serous EOC samples) and one miRNA dataset (GSE25202, *n* = 55 including 30 early relapsing patients and 25 late relapsing patients)	*KIF2C* was downregulated in OC. *KIF2C*, *STAT3* and *BUB1* were regulated by hsa-miR-494.	[194]
		Seven hub genes (*BUB1*, *KIF2C*, *NUP43*, *NDC80*, *NUF2*, *CCNB2*, and *CENPN*) were differentially expressed in platinum-resistant ovarian cancer cells; *KIF2C* is downregulated.	
	• *In vitro* methods: validated using A2780 ovarian cancer cell line, RT-PCR		
	• *In silico*: GEO dataset GSE14407 including 12 healthy controls and 12 OC patients	Expression of *CCNB1*, *CCNB2*, *BUB1B*, *CCNA2*, *KIF2C*, and *TOP2A* are associated with decreased survival and disease-free survival rates of OC.	[196]
	• *In silico*: GEO datasets GSE14407 (12 controls, 12 serous ovarian cancer epithelium samples), GSE29450 (10 controls, 10 ovarian cancer cell specimens), and GSE54388 (6 controls, 16 tumor epithelial component samples)	Expression of *CDK1*, *CDC20*, *CCNB1*, *BUB1B*, *CCNA2*, *KIF11*, *CDCA8*, *KIF2C*, *NDC80* and *TOP2A* was increased in OC.	[197]
Esophageal squamous cell carcinoma (ESCC)	• tissue: 415 surgically resected primary tumor tissues and 40 adjacent non-cancerous tissues, IHC of KIF2C	Higher KIF2C expression was associated with significantly increased risks of higher pathologic tumor status and poorer tumor differentiation. KIF2C expression in tumor tissues seems to serve as an independent prognostic marker for male (OS and DFS), but not female.	[176]
Renal cell cancer (RCC)	• *In silico*: public RNA-seq. data from TCGA; *n* = 533 KIRC	Ten genes associated with the cell cycle and p53 signaling pathway were validated as prognostic markers: *CCNA2*, *CDC20*, *CDCA8*, *GTSE1*, *KIF23*, *KIF2C*, *KIF4A*, *MELK*, *TOP2A*, and *TPX2*.	[182]
	• *In silico*: GEO dataset (GSE168845); clear cell renal cell carcinoma (ccRCC); *n* = 8, 4 ccRCC and 4 controls; UALCAN database for methylation analysis	The most enriched pathways were T cell activation and cytokine-cytokine receptor interaction. 22 hub genes related to ccRCC. KIF2C had lower methylation levels in ccRCC tissues compared with paired tumor-free kidney tissues.	[185]
	• *In silico:* TCGA, UALCAN, TIMER, and CellMiner GSCA database for methylation analysis	*KIF2C* was highly expressed in several tumors, including KIRC and KIRP, correlated with poor prognosis. *KIF2C* expression was significantly associated with TMB, MSI, MMRs, and immune checkpoint genes, and with the level of immune cell infiltration such as tumor-associated macrophage (TAM), cancer-associated fibroblasts (CAFs), myeloid-derived suppressor cells (MDSCs) and Tregs. KIF2C is also associated with DNA methylation, m6A and m7G modifications.	[105]
	• *In silico*: TCGA, GTEx database, GEPIA2, CCLE, UALCAN, CPTAC, HPA, TIMER2; analyses of various tumor entities including KIRC and KIRP	High *KIF2C* expression was significantly related to the pathological stages of KIRC and KIRP. Increased *KIF2C* is associated with poor OS and DFS.	[95]
	• *In silico:* TCGA database	Nine highly expressed KIF genes (*KIF2C*, *KIF4A*, *KIF7*, *KIF11*, *KIF14*, *KIF18A*, *KIF18B*, *KIF20A*, *KIF20B*)	[118]
		*KIF2C* gene expression was significantly related to reduced OS.	
	• *In silico:* TCGA, chromophobe renal cell carcinoma	Five hub lncRNAs (TTK, CENPE, KIF2C, BUB1, and RAD51AP1) may act as potential biomarkers for chromophobe renal cell carcinoma progression and prognosis.	[183]
Pancreatic carcinoma (PCa)	• TCGA/GEPIA analysis and PDAC tissue: 14 pairs of cancer and paracancerous tissues; RT-PCR, IHC	KIF2C expression is significantly upregulated in human PDAC tissues associated with a poor prognosis.	[99]
	• cell lines: ASPC-1 and MIA-PaCa2 (siRNA/shRNA and overexpression) and animal model with MIA-PaCa2	KIF2C promotes PDAC cell proliferation, migration, invasion, and metastasis, *in vitro* and *in vivo*. KIF2C overexpression causes a decrease in some proinflammatory factors and chemokines. In animal models, downregulation of KIF2C greatly inhibits the formation of subcutaneous tumors and lung metastasis.	
	• *In silico*: TCGA, GTEx database, GEPIA2, CCLE, UALCAN, CPTAC, HPA, TIMER2; analyses of various tumor entities including PAAD	The gene and protein expression level of KIF2C was higher in tumor tissue of PAAD. Increased levels of KIF2C expression were significantly associated with poor OS and DSF. High expression of KIF2C was significantly associated with *E2F*, *EGFR*, *MYC* and *KRAS*.	[95]
	• scRNA-seq. of 24 primary PDAC tumors and 11 control pancreases (database: CancerSCEM)	KIF2C expression is highly enriched in CD4+ T cells, fibroblasts, NK cells, and most significant in malignant cells of PDAC.	
	• *In silico*: PDAC TCGA/ GEPIA, Oncomine datasets, cBioPortal, LOGpc, TIMER, and STRING bioinformatics tools	*KIFC1/2C/4A/11/14/15/18A/18B/20B/23* were upregulated and associated with unfavorable clinical outcome. Poorer patient survival is associated with high *KIF2C* expression. *KIFC1/2C/4A/11/14/15/18A/18B/20B/23* were significantly associated with the mutation status of KRAS and TP53. *KIF2C/4A/11/14/15/16B/20A/22/23/25* were core enriched in immunologic signature. KIF genes were correlated with tumor stage and myeloid-derived suppressor cells infiltration (MDSCs).	[179]
Acute lymphoblastic leukemia (ALL)	• localization: HPA and IF in U2OS, SHIA, RH-3, ASPC1 and BXPC3	nuclear localization of KIF2C	[180]
	• *In silico* databases: GEPIA2, TISIDB, Linkdeomics, miRDB, miRanda, miRTairBase, Targetscan and miRmap, TISIDB, GSE62452 and PACA-UA	*KIF2C* mRNA expression levels were higher in pancreatic cancer tissues and significantly associated with decreased OS and DFS. *KIF2C* expression was closely related to CD4 +T cells.	
	• *In silico*: TCGA, GTEx, CCLE; 31 normal tissues, 30 tumor cell lines, and 33 tumor tissues and compared *KIF2C* expression levels in 33 cancer samples and corresponding paracancer samples and TCGA prostate adenocarcinoma (PRAD) dataset with 249 patients in the *KIF2C* high expression group and 250 patients in the *KIF2C* low expression group	*KIF2C* was significantly upregulated, associated with age, pathological stage, lymph node metastases, prostate-specific antigen, and Gleason score and significantly predicted an unfavorable prognosis. Increased *KIF2C* was significantly correlated with poor OS, DSS, DFI, and PFI.	[181]
	• tissue: tumor samples and related paracrine tissues from 14 pairs of PCa patients	*KIF2C* was involved in the cell cycle and immune response. KIF2C DNA methylation was reduced and inversely linked with KIF2C expression. *KIF2C* has a strong relationship with the TME, infiltrating cells, and immune checkpoint genes. High *KIF2C* expression was significantly resistant to a variety of MAPK signaling pathway-related inhibitors.	
Acute lymphoblastic leukemia (ALL)	• *In silico*: databases including TARGET, GSE60926 and GSE28460; all patients *n* = 134, relapse *n* = 116	*KIF2C* and *KIF18B* are overexpressed in patients with relapsed ALL compared to patients diagnosed with ALL for the first time.	[198]
	• *In vivo*: zebrafish embryos (50% sequence homology)	KIF2C is necessary for lymphopoiesis in zebrafish embryos. KIF2C is important for the maintenance and survival of hematopoietic stem cells.	
Cervical cancer	• *In silico:* 33 TCGA cancer types, TIMER 2.0, GETx, GEPIA2, UALCAN	KIF2C expression was significantly upregulated in cervical cancer tissues and cervical cancer cells. KIF2C is associated with enhanced proliferation, invasion, and migration *in vitro* and increased tumor growth *in vivo*.	[184]
	• tissue: 35 cervical squamous tissue samples, 25 cervical adenocarcinoma tissue samples, and 40 normal cervical tissues samples		
	• cell lines: cervical cancer cell lines SiHa, HeLa, C33a, and Caski and normal cervical cell line HCerEpic; shRNA used for C33a and SiHa cells, also for subcutaneous xenograft model.; vector DNA for Caski cells	KIF2C knockdown promotes the p53 signaling pathway A KIF2C and p53 double knockdown partially reversed the inhibitory influence of KIF2C silencing on cervical cancer processes.	

## Data Availability

All data generated or analyzed during this study are included in this article and its supplementary information.

## Abbreviations:

ALCAM: Activated Leukocyte Cell adhesion Molecule
ALL: acute lymphoblastic leukemia
APC: anaphase-promoting complex
ANLN: anillin actin-binding protein
ATM: Ataxia-telangiectasia mutated
BUB1B: Budding Uninhibited by Benzimidazoles 1 Homolog Beta
CDCA8: cell division cycle associated 8
CENPM: centromere protein M
CIN: chromosomal instability
ccRCC: clear cell renal cell carcinoma
CTPS1: CTP Synthase 1
CCNA2: cyclin A2
CCNB1: cyclin B1
Cdk1: cyclin-dependent kinase 1
CLASP: cytoplasmic linker-associated protein
DSF: disease-free survival
TOP2A: DNA topoisomerase II α
MYBL2: Myb-related protein B
DSBs: double-strand breaks
DOX: doxorubicin
E2F1: E2F transcription factor 1
EMT: epithelial-to-mesenchymal transition
ER: estrogen
ECV: extracellular environment
ECM: extracellular matrix
ERK: extracellular signal-regulated kinase
FAK: focal adhesion kinase
FA: focal adhesion
HCC: hepatocellular carcinoma
HDAC4: histone deacetylase 4
HNF4A: Hepatocyte Nuclear Factor 4 Alpha
HER2: human epidermal growth factor receptor 2
IL: interleukin
KIRC: kidney renal clear cell carcinoma
KNSTRN: kinetochore-localized astrin/SPAG5 binding protein
K-Ras: Kirsten rat sarcoma virus
LSCC: laryngeal squamous cell carcinoma
mTORC1: mammalian target of rapamycin complex 1
MELK: maternal embryonic leucine zipper kinase
miRNAs: micro RNAs
MAPs: microtubule associated proteins
TIP150: tracking protein of 150 KDa
MTOCs: microtubule organizing centers
MT: microtubule
MAPK: mitogen-activated protein kinase
MAD2L1: mitotic arrest deficient 2 like 1
NPC: nasopharyngeal carcinoma
NSCLC: non-small cell lung cancer
lncRNAs: long non-coding RNAs
NFKB1: Nuclear Factor Kappa B Subunit 1
NuMa: nuclear mitotic apparatus
NuSAP: nucleolar spindle-associated protein
OS: overall survival
PAK1: p21-activated kinase 1
PCa: pancreatic cancer
PDAC: pancreatic ductal adenocarcinoma
PDOs: patient-derived tumor organoids
Plk1: Polo-like kinase 1
PARPBP: poly (ADP-ribose) polymerase 1 binding protein
PARP: poly (ADP-ribose) polymerase 1
PFS: progression-free survival
PST: postoperative survival time
PRC1: protein regulator of cytokinesis 1
PKM2: pyruvate kinase M2
PRISMA: preferred reporting items for Systematic Reviews and Meta-Analyses
RAC1: Rac Family Small GTPase 1
RAD51AP1: RAD51 Associated Protein 1
RCC: renal cell cancer
RITA: RBP-J interacting and tubulin-associated protein
FAM134B: Reticulophagy Regulator 1
RPE-1: retinal pigment epithelial cells
scRNA-seq: single-cell RNA-sequencing
STAT3: signal transducer and activator of transcription 3
SMAD4: SMAD Family Member 4
BIRC5: survivin
TBC1D7: Tre2-Bub2-Cdc16 domain family member 7
TCGA: The Cancer Genome Atlas
TNBC: triple negative breast cancer
TPX2: Targeting protein for Xklp2
TBC1D7: Tre2-Bub2-Cdc16 domain family member 7
TME: tumor microenvironment
TP53: Tumor Protein P53
T stage: tumor staging
TILs: tumor-infiltrating lymphocytes
UBE2C: ubiquitin conjugating enzyme E2C
WS: weighted score
Wnt: wingless
XMAP215: Xenopus microtubule-associated protein
