# Supplementary material for: KIF2C/MCAK a prognostic biomarker and its oncogenic potential in malignant progression, and prognosis of cancer patients: a systematic review and meta-analysis as biomarker
Source: Crit Rev Clin Lab Sci. Author manuscript; Available in PMC 2025 Feb 12. (PMC11815995; doi:10.1080/10408363.2024.2309933)
Supplement: Supplement Table 1 [file NIHMS2046882-supplement-Supplement_Table_1.docx]

PubMed/NCBI (last update of the search strategy on May 15, 2023):

| Search: | Input: | No. of references: |
| --- | --- | --- |
| 1# | Search: **(KIF2C gastric cancer) OR (MCAK gastric cancer)**  ("KIF2C"[All Fields] AND ("stomach neoplasms"[MeSH Terms] OR ("stomach"[All Fields] AND "neoplasms"[All Fields]) OR "stomach neoplasms"[All Fields] OR ("gastric"[All Fields] AND "cancer"[All Fields]) OR "gastric cancer"[All Fields])) OR ("MCAK"[All Fields] AND ("stomach neoplasms"[MeSH Terms] OR ("stomach"[All Fields] AND "neoplasms"[All Fields]) OR "stomach neoplasms"[All Fields] OR ("gastric"[All Fields] AND "cancer"[All Fields]) OR "gastric cancer"[All Fields]))  **Translations**  **gastric cancer:** "stomach neoplasms"[MeSH Terms] OR ("stomach"[All Fields] AND "neoplasms"[All Fields]) OR "stomach neoplasms"[All Fields] OR ("gastric"[All Fields] AND "cancer"[All Fields]) OR "gastric cancer"[All Fields] | 6 |
| #2 | Search: **(KIF2C colorectal cancer) OR (MCAK colorectal cancer)**  ("KIF2C"[All Fields] AND ("colorectal neoplasms"[MeSH Terms] OR ("colorectal"[All Fields] AND "neoplasms"[All Fields]) OR "colorectal neoplasms"[All Fields] OR ("colorectal"[All Fields] AND "cancer"[All Fields]) OR "colorectal cancer"[All Fields])) OR ("MCAK"[All Fields] AND ("colorectal neoplasms"[MeSH Terms] OR ("colorectal"[All Fields] AND "neoplasms"[All Fields]) OR "colorectal neoplasms"[All Fields] OR ("colorectal"[All Fields] AND "cancer"[All Fields]) OR "colorectal cancer"[All Fields]))  **Translations**  **colorectal cancer:** "colorectal neoplasms"[MeSH Terms] OR ("colorectal"[All Fields] AND "neoplasms"[All Fields]) OR "colorectal neoplasms"[All Fields] OR ("colorectal"[All Fields] AND "cancer"[All Fields]) OR "colorectal cancer"[All Fields] | 8 |
| #3 | Search: **(KIF2C breast cancer) OR (MCAK breast cancer)**  ("KIF2C"[All Fields] AND ("breast neoplasms"[MeSH Terms] OR ("breast"[All Fields] AND "neoplasms"[All Fields]) OR "breast neoplasms"[All Fields] OR ("breast"[All Fields] AND "cancer"[All Fields]) OR "breast cancer"[All Fields])) OR ("MCAK"[All Fields] AND ("breast neoplasms"[MeSH Terms] OR ("breast"[All Fields] AND "neoplasms"[All Fields]) OR "breast neoplasms"[All Fields] OR ("breast"[All Fields] AND "cancer"[All Fields]) OR "breast cancer"[All Fields]))  **Translations**  **breast cancer:** "breast neoplasms"[MeSH Terms] OR ("breast"[All Fields] AND "neoplasms"[All Fields]) OR "breast neoplasms"[All Fields] OR ("breast"[All Fields] AND "cancer"[All Fields]) OR "breast cancer"[All Fields] | 34 |
| Exclusion: | - Articles used mice cell line mCaK - KIF2C/MCAK as secondary target - Other cancer entity studies referencing breast cancer. - Interactome of NEK5 in breast cancer cells | 5 |
| #4 | Search: **(((KIF2C Non-small cell lung cancer) OR (MCAK Non-small cell lung cancer)) OR (KIF2C NSCLC)) OR (MCAK NSCLC)**  ("KIF2C"[All Fields] AND ("carcinoma, non small cell lung"[MeSH Terms] OR ("carcinoma"[All Fields] AND "non small cell"[All Fields] AND "lung"[All Fields]) OR "non-small-cell lung carcinoma"[All Fields] OR ("non"[All Fields] AND "small"[All Fields] AND "cell"[All Fields] AND "lung"[All Fields] AND "cancer"[All Fields]) OR "non small cell lung cancer"[All Fields])) OR ("MCAK"[All Fields] AND ("carcinoma, non small cell lung"[MeSH Terms] OR ("carcinoma"[All Fields] AND "non small cell"[All Fields] AND "lung"[All Fields]) OR "non-small-cell lung carcinoma"[All Fields] OR ("non"[All Fields] AND "small"[All Fields] AND "cell"[All Fields] AND "lung"[All Fields] AND "cancer"[All Fields]) OR "non small cell lung cancer"[All Fields])) OR ("KIF2C"[All Fields] AND ("carcinoma, non small cell lung"[MeSH Terms] OR ("carcinoma"[All Fields] AND "non small cell"[All Fields] AND "lung"[All Fields]) OR "non-small-cell lung carcinoma"[All Fields] OR "nsclc"[All Fields] OR "nsclc s"[All Fields] OR "nsclcs"[All Fields])) OR ("MCAK"[All Fields] AND ("carcinoma, non small cell lung"[MeSH Terms] OR ("carcinoma"[All Fields] AND "non small cell"[All Fields] AND "lung"[All Fields]) OR "non-small-cell lung carcinoma"[All Fields] OR "nsclc"[All Fields] OR "nsclc s"[All Fields] OR "nsclcs"[All Fields]))  **Translations**  **Non-small cell lung cancer:** "carcinoma, non-small-cell lung"[MeSH Terms] OR ("carcinoma"[All Fields] AND "non-small-cell"[All Fields] AND "lung"[All Fields]) OR "non-small-cell lung carcinoma"[All Fields] OR ("non"[All Fields] AND "small"[All Fields] AND "cell"[All Fields] AND "lung"[All Fields] AND "cancer"[All Fields]) OR "non small cell lung cancer"[All Fields]  **NSCLC:** "carcinoma, non-small-cell lung"[MeSH Terms] OR ("carcinoma"[All Fields] AND "non-small-cell"[All Fields] AND "lung"[All Fields]) OR "non-small-cell lung carcinoma"[All Fields] OR "nsclc"[All Fields] OR "nsclc's"[All Fields] OR "nsclcs"[All Fields] | 8 |
| #5 | Search: **(((KIF2C Hepatocellular carcinoma) OR (MCAK Hepatocellular carcinoma)) OR (KIF2C HCC)) OR (MCAK HCC)**  ("KIF2C"[All Fields] AND ("carcinoma, hepatocellular"[MeSH Terms] OR ("carcinoma"[All Fields] AND "hepatocellular"[All Fields]) OR "hepatocellular carcinoma"[All Fields] OR ("hepatocellular"[All Fields] AND "carcinoma"[All Fields]))) OR ("MCAK"[All Fields] AND ("carcinoma, hepatocellular"[MeSH Terms] OR ("carcinoma"[All Fields] AND "hepatocellular"[All Fields]) OR "hepatocellular carcinoma"[All Fields] OR ("hepatocellular"[All Fields] AND "carcinoma"[All Fields]))) OR ("KIF2C"[All Fields] AND "HCC"[All Fields]) OR ("MCAK"[All Fields] AND "HCC"[All Fields])  **Translations**  **Hepatocellular carcinoma:** "carcinoma, hepatocellular"[MeSH Terms] OR ("carcinoma"[All Fields] AND "hepatocellular"[All Fields]) OR "hepatocellular carcinoma"[All Fields] OR ("hepatocellular"[All Fields] AND "carcinoma"[All Fields]) | 22 |
| Exclusion: | - Articles used mice mammary carcinoma “mCa-K” - Retracted articles due to scientific flaws | 3 |
| #6 | Search: **(KIF2C glioma) OR (MCAK glioma)**  ("KIF2C"[All Fields] AND ("glioma"[MeSH Terms] OR "glioma"[All Fields] OR "gliomas"[All Fields] OR "glioma s"[All Fields])) OR ("MCAK"[All Fields] AND ("glioma"[MeSH Terms] OR "glioma"[All Fields] OR "gliomas"[All Fields] OR "glioma s"[All Fields]))  **Translations**  **glioma**: "glioma"[MeSH Terms] OR "glioma"[All Fields] OR "gliomas"[All Fields] OR "glioma's"[All Fields] | 7 |
| Exclusion: | - Studies not including any types of gliomas or references to KIF2C/MCAK | 2 |
| #7 | Search: **(KIF2C endometrial cancer) OR (MCAK endometrial cancer)**  ("KIF2C"[All Fields] AND ("endometrial neoplasms"[MeSH Terms] OR ("endometrial"[All Fields] AND "neoplasms"[All Fields]) OR "endometrial neoplasms"[All Fields] OR ("endometrial"[All Fields] AND "cancer"[All Fields]) OR "endometrial cancer"[All Fields])) OR ("MCAK"[All Fields] AND ("endometrial neoplasms"[MeSH Terms] OR ("endometrial"[All Fields] AND "neoplasms"[All Fields]) OR "endometrial neoplasms"[All Fields] OR ("endometrial"[All Fields] AND "cancer"[All Fields]) OR "endometrial cancer"[All Fields]))  **Translations**  **endometrial cancer:** "endometrial neoplasms"[MeSH Terms] OR ("endometrial"[All Fields] AND "neoplasms"[All Fields]) OR "endometrial neoplasms"[All Fields] OR ("endometrial"[All Fields] AND "cancer"[All Fields]) OR "endometrial cancer"[All Fields] | 5 |
| Exclusion | - Study not including endometrial cancer | 1 |
| #8 | Search: **(((KIF2C ovarian cancer) OR (MCAK ovarian cancer)) OR (KIF2C OC)) OR (MCAK OC)**  ("KIF2C"[All Fields] AND ("ovarian neoplasms"[MeSH Terms] OR ("ovarian"[All Fields] AND "neoplasms"[All Fields]) OR "ovarian neoplasms"[All Fields] OR ("ovarian"[All Fields] AND "cancer"[All Fields]) OR "ovarian cancer"[All Fields])) OR ("MCAK"[All Fields] AND ("ovarian neoplasms"[MeSH Terms] OR ("ovarian"[All Fields] AND "neoplasms"[All Fields]) OR "ovarian neoplasms"[All Fields] OR ("ovarian"[All Fields] AND "cancer"[All Fields]) OR "ovarian cancer"[All Fields])) OR ("KIF2C"[All Fields] AND "OC"[All Fields]) OR ("MCAK"[All Fields] AND "OC"[All Fields])  **Translations**  **ovarian cancer:** "ovarian neoplasms"[MeSH Terms] OR ("ovarian"[All Fields] AND "neoplasms"[All Fields]) OR "ovarian neoplasms"[All Fields] OR ("ovarian"[All Fields] AND "cancer"[All Fields]) OR "ovarian cancer"[All Fields] | 9 |
| Exclusion | - Study not including endometrial cancer - Studies using MCa-K cells - Study involving cervical cancer not ovarian cancer | 5 |
| #9 | Search: **(((Kidney renal clear cell carcinoma MCAK) OR (Kidney renal clear cell carcinoma KIF2C)) OR (KIRC MCAK)) OR (KIRC KIF2C)**  (("kidney"[MeSH Terms] OR "kidney"[All Fields] OR "kidneys"[All Fields] OR "kidney s"[All Fields]) AND ("carcinoma, renal cell"[MeSH Terms] OR ("carcinoma"[All Fields] AND "renal"[All Fields] AND "cell"[All Fields]) OR "renal cell carcinoma"[All Fields] OR ("renal"[All Fields] AND "clear"[All Fields] AND "cell"[All Fields] AND "carcinoma"[All Fields]) OR "renal clear cell carcinoma"[All Fields]) AND "MCAK"[All Fields]) OR (("kidney"[MeSH Terms] OR "kidney"[All Fields] OR "kidneys"[All Fields] OR "kidney s"[All Fields]) AND ("carcinoma, renal cell"[MeSH Terms] OR ("carcinoma"[All Fields] AND "renal"[All Fields] AND "cell"[All Fields]) OR "renal cell carcinoma"[All Fields] OR ("renal"[All Fields] AND "clear"[All Fields] AND "cell"[All Fields] AND "carcinoma"[All Fields]) OR "renal clear cell carcinoma"[All Fields]) AND "KIF2C"[All Fields]) OR ("KIRC"[All Fields] AND "MCAK"[All Fields]) OR ("KIRC"[All Fields] AND "KIF2C"[All Fields])  **Translations**  **Kidney:** "kidney"[MeSH Terms] OR "kidney"[All Fields] OR "kidneys"[All Fields] OR "kidney's"[All Fields]  renal clear cell carcinoma: "carcinoma, renal cell"[MeSH Terms] OR ("carcinoma"[All Fields] AND "renal"[All Fields] AND "cell"[All Fields]) OR "renal cell carcinoma"[All Fields] OR ("renal"[All Fields] AND "clear"[All Fields] AND "cell"[All Fields] AND "carcinoma"[All Fields]) OR "renal clear cell carcinoma"[All Fields] | 3 |
| 10# | Search**: ((((esophageal squamous cell carcinoma KIF2C) OR (esophageal squamous cell carcinoma MCAK)) OR (ESCC KIF2C)) OR (ESCC MCAK)**  (("esophageal squamous cell carcinoma"[MeSH Terms] OR ("esophageal"[All Fields] AND "squamous"[All Fields] AND "cell"[All Fields] AND "carcinoma"[All Fields]) OR "esophageal squamous cell carcinoma"[All Fields]) AND "KIF2C"[All Fields]) OR (("esophageal squamous cell carcinoma"[MeSH Terms] OR ("esophageal"[All Fields] AND "squamous"[All Fields] AND "cell"[All Fields] AND "carcinoma"[All Fields]) OR "esophageal squamous cell carcinoma"[All Fields]) AND "MCAK"[All Fields]) OR ("ESCC"[All Fields] AND "KIF2C"[All Fields]) OR ("ESCC"[All Fields] AND "MCAK"[All Fields])  **Translations**  **esophageal squamous cell carcinoma:** "esophageal squamous cell carcinoma"[MeSH Terms] OR ("esophageal"[All Fields] AND "squamous"[All Fields] AND "cell"[All Fields] AND "carcinoma"[All Fields]) OR "esophageal squamous cell carcinoma"[All Fields] | 2 |
| 11# | **Search: (((KIF2C Kidney renal clear cell carcinoma) OR (MCAK Kidney renal clear cell carcinoma) OR (KIF2C Renal cell carcinoma) OR (MCAK Renal cell carcinoma)) OR (KIF2C KIRC)) OR (MCAK KIRC)**  ("KIF2C"[All Fields] AND ("kidney"[MeSH Terms] OR "kidney"[All Fields] OR "kidneys"[All Fields] OR "kidney s"[All Fields]) AND ("carcinoma, renal cell"[MeSH Terms] OR ("carcinoma"[All Fields] AND "renal"[All Fields] AND "cell"[All Fields]) OR "renal cell carcinoma"[All Fields] OR ("renal"[All Fields] AND "clear"[All Fields] AND "cell"[All Fields] AND "carcinoma"[All Fields]) OR "renal clear cell carcinoma"[All Fields])) OR ("MCAK"[All Fields] AND ("kidney"[MeSH Terms] OR "kidney"[All Fields] OR "kidneys"[All Fields] OR "kidney s"[All Fields]) AND ("carcinoma, renal cell"[MeSH Terms] OR ("carcinoma"[All Fields] AND "renal"[All Fields] AND "cell"[All Fields]) OR "renal cell carcinoma"[All Fields] OR ("renal"[All Fields] AND "clear"[All Fields] AND "cell"[All Fields] AND "carcinoma"[All Fields]) OR "renal clear cell carcinoma"[All Fields])) OR ("KIF2C"[All Fields] AND "KIRC"[All Fields]) OR ("MCAK"[All Fields] AND "KIRC"[All Fields])  **Translations**  **Kidney:** "kidney"[MeSH Terms] OR "kidney"[All Fields] OR "kidneys"[All Fields] OR "kidney's"[All Fields]  renal clear cell carcinoma: "carcinoma, renal cell"[MeSH Terms] OR ("carcinoma"[All Fields] AND "renal"[All Fields] AND "cell"[All Fields]) OR "renal cell carcinoma"[All Fields] OR ("renal"[All Fields] AND "clear"[All Fields] AND "cell"[All Fields] AND "carcinoma"[All Fields]) OR "renal clear cell carcinoma"[All Fields] | 4 |
| 12# | **Search: (((KIF2C PDAC) OR (MCAK PDAC)) OR (KIF2C Pancreatic Ductal Adenocarcinoma)) OR (MCAK Pancreatic Ductal Adenocarcinoma)**  ("KIF2C"[All Fields] AND "PDAC"[All Fields]) OR ("MCAK"[All Fields] AND "PDAC"[All Fields]) OR ("KIF2C"[All Fields] AND ("pancreas"[MeSH Terms] OR "pancreas"[All Fields] OR "pancreatic"[All Fields] OR "pancreatitides"[All Fields] OR "pancreatitis"[MeSH Terms] OR "pancreatitis"[All Fields]) AND "Ductal"[All Fields] AND ("adenocarcinoma"[MeSH Terms] OR "adenocarcinoma"[All Fields] OR "adenocarcinomas"[All Fields] OR "adenocarcinoma s"[All Fields])) OR ("MCAK"[All Fields] AND ("pancreas"[MeSH Terms] OR "pancreas"[All Fields] OR "pancreatic"[All Fields] OR "pancreatitides"[All Fields] OR "pancreatitis"[MeSH Terms] OR "pancreatitis"[All Fields]) AND "Ductal"[All Fields] AND ("adenocarcinoma"[MeSH Terms] OR "adenocarcinoma"[All Fields] OR "adenocarcinomas"[All Fields] OR "adenocarcinoma s"[All Fields]))  **Translations**  **Pancreatic:** "pancreas"[MeSH Terms] OR "pancreas"[All Fields] OR "pancreatic"[All Fields] OR "pancreatitides"[All Fields] OR "pancreatitis"[MeSH Terms] OR "pancreatitis"[All Fields]  Adenocarcinoma: "adenocarcinoma"[MeSH Terms] OR "adenocarcinoma"[All Fields] OR "adenocarcinomas"[All Fields] OR "adenocarcinoma's"[All Fields]  Pancreatic: "pancreas"[MeSH Terms] OR "pancreas"[All Fields] OR "pancreatic"[All Fields] OR "pancreatitides"[All Fields] OR "pancreatitis"[MeSH Terms] OR "pancreatitis"[All Fields]  Adenocarcinoma: "adenocarcinoma"[MeSH Terms] OR "adenocarcinoma"[All Fields] OR "adenocarcinomas"[All Fields] OR "adenocarcinoma's"[All Fields] | 1 |
| 13# | **Search: (((PAAD KIF2C) OR (PAAD MCAK)) OR (pancreatic cancer KIF2C)) OR (pancreatic cancer MCAK)**  ("PAAD"[All Fields] AND "KIF2C"[All Fields]) OR ("PAAD"[All Fields] AND "MCAK"[All Fields]) OR (("pancreatic neoplasms"[MeSH Terms] OR ("pancreatic"[All Fields] AND "neoplasms"[All Fields]) OR "pancreatic neoplasms"[All Fields] OR ("pancreatic"[All Fields] AND "cancer"[All Fields]) OR "pancreatic cancer"[All Fields]) AND "KIF2C"[All Fields]) OR (("pancreatic neoplasms"[MeSH Terms] OR ("pancreatic"[All Fields] AND "neoplasms"[All Fields]) OR "pancreatic neoplasms"[All Fields] OR ("pancreatic"[All Fields] AND "cancer"[All Fields]) OR "pancreatic cancer"[All Fields]) AND "MCAK"[All Fields])  **Translations**  **pancreatic cancer:** "pancreatic neoplasms"[MeSH Terms] OR ("pancreatic"[All Fields] AND "neoplasms"[All Fields]) OR "pancreatic neoplasms"[All Fields] OR ("pancreatic"[All Fields] AND "cancer"[All Fields]) OR "pancreatic cancer"[All Fields] | 6 |
| Exclusion | - Study only including KIF2C in the reference list. - Study about other cancer entities. | 1 |

Preprint databases (BioRxiv and Researchsquare):

| Search: | Input: | No. of references: |
| --- | --- | --- |
| **Researchsquare #1** | Search: **(KIF2C) OR (MCAK)**  ("KIF2C"[All Fields] OR ("MCAK"[All Fields]))  (Period: 01.01.2021 – 01.03.2023) | 12 |
| Exclusion: | - Studies found a correlation between a target gene and KIF2C (#1) - Studies analyzed KIF2Cs role in male infertility (#2) - Studies were already published in peer-reviewed journals (#9, published articles were integrated in the manuscript) | 12 |
| **bioRxiv/medRxiv #1** | Search: **(KIF2C) OR (MCAK)**  ("KIF2C"[All Fields] OR ("MCAK"[All Fields]))  (Period: 01.01.2021 – 01.03.2023) | 136 |
| Exclusion: | - Studies found a correlation between a target gene and KIF2C. - Studies in other species e.g., budding yeast and mouse oocytes. - Articles mentioning KIF2C only in the literature list. - Studies investigated the molecular mechanisms of KIF2C in spindle assembly/regulation. - Studies investigate KIF2Cs role in other diseases. - Studies were already published in peer-reviewed journals (#2, published articles were integrated in the manuscript). | 133 |
